# Identification and characterization of QTLs for brown planthopper resistance from wild rice, *Oryza nivara* (Sharma et Shastry)

**DOI:** 10.1270/jsbbs.25038

**Published:** 2025-10-25

**Authors:** Hoang Nam Nguyen, Cuong Dinh Nguyen, Sachiyo Sanada-Morimura, Shao-Hui Zheng, Daisuke Fujita

**Affiliations:** 1 The United Graduate School of Agricultural Sciences, Kagoshima University, 1-21-24 Korimoto, Kagoshima 890-0065, Japan; 2 Biotechnology Department, College of Food Industry, 101B Le Huu Trac Street, Son Tra District, Da Nang City 550000, Vietnam; 3 Agro-Environment Research Division, Kyushu Okinawa Agricultural Research Center, NARO, 2421 Suya, Koshi, Kumamoto 861-1192, Japan; 4 Faculty of Agriculture, Saga University, 1 Honjo-machi, Saga 840-8502, Japan

**Keywords:** NIL, BPH resistance, resistance mechanisms, *Nilaparvata lugens*

## Abstract

The brown planthopper (*Nilaparvata lugens* (Stål); BPH) is a serious insect pest of rice (*Oryza sativa* L.). Host plant resistance is an effective means of controlling this pest; up to date at least 46 BPH resistance genes have been identified. However, BPH can overcome resistance conferred by single resistance genes. Therefore, it is necessary to detect new and durable resistance genes. Here, we identified quantitative trait loci (QTLs) for BPH resistance from the wild rice *Oryza nivara* (Sharma et Shastry) IRGC 89073. Using backcrossed populations derived from IRGC 89073 × the susceptible ‘Taichung 65’, we detected two resistance QTLs, *qBPH4* on chromosome 4 and *qBPH11* on chromosome 11. *qBPH11* was validated in the BC_3_F_2_ population. A near-isogenic line carrying *qBPH11* showed significant resistance to BPH in antibiosis, antixenosis, MSST, and honeydew tests. These results suggest that IRGC 89073 harbors valuable genetic resources that could enhance BPH resistance in rice breeding programs, particularly through pyramiding strategies using marker-assisted selection.

## Introduction

Rice (*Oryza sativa* L.) is a staple food for more than half of the world’s population. However, its production is threatened by the brown planthopper (BPH), *Nilaparvata lugens* (Stål), a major insect pest that feeds on the phloem sap and significantly reduces grain yield. In severe infestations, “hopperburn” occurs, when rice plants dry out and die. BPH can also transmit *Rice grassy stunt virus* (RGSV) and *Rice ragged stunt virus* (RRSV), which can further decrease yield (Cabauatan *et al.* 2009). Severe yield losses caused by BPH outbreaks have been reported in recent years in several rice-producing countries, including India, Indonesia, China, Japan, Vietnam, Korea, and Bangladesh (Sogawa 2015). While chemical pesticides are the primary method used to control BPH, their use poses both economic and environmental challenges. Excessive use of insecticides not only increases production costs but also harms natural enemies, leading to ecological imbalances and the resurgence of more virulent biotypes of BPH (Tanaka *et al.* 2000). In contrast, using host plant resistance provides a more sustainable and environmentally friendly strategy for effective and long-term BPH management.

Since the late 1960s, scientists have been screening and identifying BPH resistance germplasm from wild rice species and cultivars across Southeast Asia (Brar and Khush 1997). At least 46 genes associated with BPH resistance (designated *BPH1* to *BPH46*) have been identified (Fujita *et al.* 2013, Hu *et al.* 2016, Yan *et al.* 2023). The derivation of over half of these genes from wild rice species suggests their rich genetic diversity and potential as a valuable source of new alleles for BPH resistance (Brar and Khush 1997). However, only a small number confers broad-spectrum effectiveness in monogenic rice lines due to the emergence of new BPH biotypes in Asia (Fujii *et al.* 2021, Nguyen *et al.* 2019). This evidence highlights the vulnerability of single resistance genes to BPH. Therefore, identifying new durable resistance genes and using them in pyramiding breeding programs are necessary to ensure stable rice production.

*O. sativa* was domesticated from *Oryza rufipogon* in Asia. *O. rufipogon* includes both perennial and annual forms. The annual form was previously classified as a separate species, *Oryza nivara* (Sharma et Shastry). In this study, we continue to use the name *O. nivara* to refer to the annual form, in line with earlier classifications and to maintain consistency with previous studies. Both forms are distributed mainly across South and Southeast Asia (Grillo *et al.* 2009). Several studies have identified BPH resistance genes derived from *O. rufipogon*, namely *BPH27*, *BPH29*, *BPH35*, *BPH36*, *BPH38*, and *BPH41* (Huang *et al.* 2013, Li *et al.* 2019, Wang *et al.* 2015, 2022, Yang *et al.* 2020, Zhang *et al.* 2020) —and from *O. nivara*: *BPH34*, *BPH39*(t), *BPH40*(t) and *BPH45* (Akanksha *et al.* 2019, Kumar *et al.* 2018, Li *et al.* 2023). These findings highlight the importance of these wild species as valuable sources of genetic resistance.

In this study, we focused on the resistant QTLs, *qBPH11* and *qBPH4*, in an accession of *O. nivara*, IRGC 89073 and validated their resistance through QTL analysis, providing theoretical insights for BPH resistance breeding. We previously found that IRGC 89073 has strong resistance to the highly virulent Koshi-2013 BPH population. To reveal the genetic basis of this resistance, we carried out quantitative trait locus (QTL) analysis using backcrossed populations derived from a cross between *O. sativa* ‘Taichung 65’ (T65) and IRGC 89073. We validated and characterized one QTL in a near-isogenic line (NIL). The findings provide valuable genetic information for breeding BPH-resistant rice cultivars through marker-assisted selection.

## Materials and Methods

### Plant materials

We crossed *O. nivara* IRGC 89073 from Lao PDR (provided by IRRI) with the susceptible T65, and backcrossed the F_1_ plants to T65. We evaluated BC_1_F_1_ plants for resistance to the highly virulent Koshi-2013 BPH population (Fujii *et al.* 2021). Resistant plants were further backcrossed to T65 to generate a BC_2_F_1_ population. We evaluated 47 BC_2_F_1_ plants (from the BC_1_F_1_ plant with the highest resistance level) for resistance to BPH and used them for QTL analysis.

**Fig. 1.**
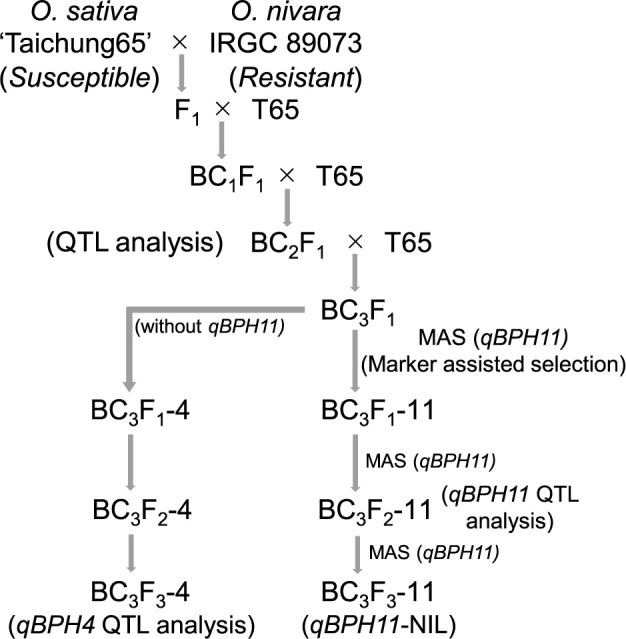
Breeding schemes for development of segregating populations and near-isogenic line derived from ‘Taichung 65’ × IRGC 89073. We named the populations for *qBPH11* BC_3_F_1_-11, BC_3_F_2_-11 and BC_3_F_3_-11; and for *qBPH4* BC_3_F_1_-4, BC_3_F_2_-4 and BC_3_F_3_-4.

We crossed nine BC_2_F_1_ plants with BPH resistance in the antibiosis test with T65 to produce BC_3_F_1_ plants. Among these plants, we selected one plant carrying *qBPH11* (named BC_3_F_1_-11) by marker-assisted selection to generate a BC_3_F_2_-11 population for validating the QTL. From this population, we selected a plant homozygous for *qBPH11* to develop a NIL at BC_3_F_3_-11 and characterized it. To detect any additional loci associated with BPH resistance, we selected a BC_3_F_1_ plant without the *qBPH11* (named BC_3_F_1_-4) and self-pollinated it to produce BC_3_F_2_-4 and BC_3_F_3_-4 populations for QTL analysis (Fig. 1).

### DNA extraction and genotyping

Total DNA of the backcrossed populations and parents was extracted by using the potassium acetate method (Dellaporta *et al.* 1983). We used polymerase chain reaction and agarose gel electrophoresis, as described in a previous study (Nguyen *et al.* 2019) to genotype the backcrossed populations using SSR markers. We tested 384 SSR markers distributed across all 12 rice chromosomes for polymorphism between donor parent IRGC 89073 and recurrent parent T65 (Supplemental Table 1). We genotyped 47 BC_2_F_1_, 154 BC_3_F_2_-11, and 132 BC_3_F_2_-4 plants by markers polymorphic between parents (McCouch *et al.* 2002, Temnykh *et al.* 2001).

### BPH populations used to evaluate plant resistance

We evaluated resistance to two BPH populations. The Hadano-1966 population, collected from Hadano City in Kanagawa Prefecture in 1966, was captured before the release of rice cultivars with BPH resistance and has weak virulence. The Koshi-2013 population, collected from Koshi City in Kumamoto Prefecture in 2013, has overcome the *BPH1* and *BPH2* resistance genes (Fujii *et al.* 2021). Both populations were maintained on the susceptible *japonica* ‘Reiho’ at 25°C under a 16-h light/8-h dark cycle at the National Agriculture and Food Research Organization. Both populations have been maintained on T65 under the same conditions at Saga University.

### Modified seedbox screening test

To evaluate BPH resistance in the NIL and BC_3_F_3_-4 populations, we conducted a modified seedbox screening test (MSST) following the method of Horgan *et al.* (2015). We sowed 25 seeds in a single row within a plastic tray (23.0 cm × 30.0 cm × 2.5 cm), along with three rows of the susceptible T65 and one row of the resistant IRGC 89073, at a row spacing of 2.5 cm. The positions of all rows were randomized. Seven days after sowing, seedlings in each row were thinned to 20 plants and then infested with second- and third-instar BPH nymphs at a density of 20 insects per plant. When all T65 plants had died, the MSST damage score was recorded using the standard evaluation system for rice (IRRI 2014).

### Honeydew test

We performed the honeydew test of Heinrichs *et al.* (1985) with modifications to evaluate the effects of the NIL and the BC_3_F_2_-11 population. We grew lines individually in 215-mL plastic cups with five replications. At 30 days, each plant was enclosed in an inverted transparent plastic cup containing filter paper stained with 0.1% bromocresol green. The filter paper changes from yellow-orange to blue upon contact with honeydew excreted by BPH. Before infestation, Hadano-1966 BPHs were starved for 1 h. Each plant was then infested with two medium-abdomen brachypterous adult female BPHs. After 24 h, the filter papers were collected and the honeydew area was measured in ImageJ v. 1.53a software (National Institutes of Health, Bethesda, MD, USA; https://rsb.info.nih.gov/ij).

### Antibiosis test

We conducted antibiosis tests following the method of Tanaka (2000) to see the effects of QTLs associated with BPH resistance. Lines carrying a resistance QTL, along with the parental lines, were individually sown in 215-mL plastic cups, with five replications each. At 30 days, the plants were enclosed in transparent plastic cups and infested with five thin-abdomen brachypterous female BPHs. Adult mortality was determined by calculating the percentage of dead BPHs at 5 days after infestation (DAI).

### Antixenosis test

One plant of NIL and one T65 plant were grown together in a 215-mL plastic cup, with five replications. At 30 days, the plants were enclosed in plastic tubes fitted with ventilators. Inside each tube, 20 second-instar BPH nymphs were released. The number of insects that settled on each plant was recorded daily up to 5 DAI. The percentage of insects that settled on each plant was used to determine the level of antixenosis (Nguyen *et al.* 2021).

### Tolerance test

We used the tolerance test of Heinrichs *et al.* (1985). Individual plants of NIL, T65, and IRGC 89073 were sown in 1-L plastic cups with three replications. At 45 days, each plant was enclosed in a plastic tube with ventilation, and 100 second- and third-instar BPH nymphs were placed in each tube. Another plants of NIL, T65, and IRGC 89073 plants covered with a plastic tube were maintained as controls without infestation. When the susceptible control T65 was completely wilted, the plants were cut at the soil surface and weighed. The percentage of plant fresh weight loss (PFWL), used as an inverse measure of tolerance, was calculated as:


$$\text{PFWL (\%) = }\frac{\text{Fresh weight of control plants – Fresh weight of infested plants}}{\text{Fresh weight of control plants}}\text{ × 100\%}$$

### QTL analysis

QTL analysis of BC_2_F_1_, BC_3_F_2_-11, and BC_3_F_2_-4 populations using genotypic and phenotypic data in Windows QTL Cartographer v. 2.5 software estimated QTLs by interval mapping (IM) and composite interval mapping (CIM) methods (Wang *et al.* 2012). The optimal logarithm of odds (LOD) threshold was used to determine the presence of QTLs at threshold values of 2.5 for BC_2_F_1_, 2.6 for BC_3_F_2_-11, and 3.9 for BC_3_F_2_-4.

### Statistical analysis

The mean values of BPH resistance of the NIL were compared by one-way ANOVA. The MSST damage score, honeydew area, adult mortality, plant tolerance and antixenosis effect were compared among the NIL and parental lines by Dunnett’s test and the Tukey–Kramer test in R v. 4.3.2 software.

## Results

### QTL detection for BPH resistance in BC_2_F_1_ population

**Fig. 2.**
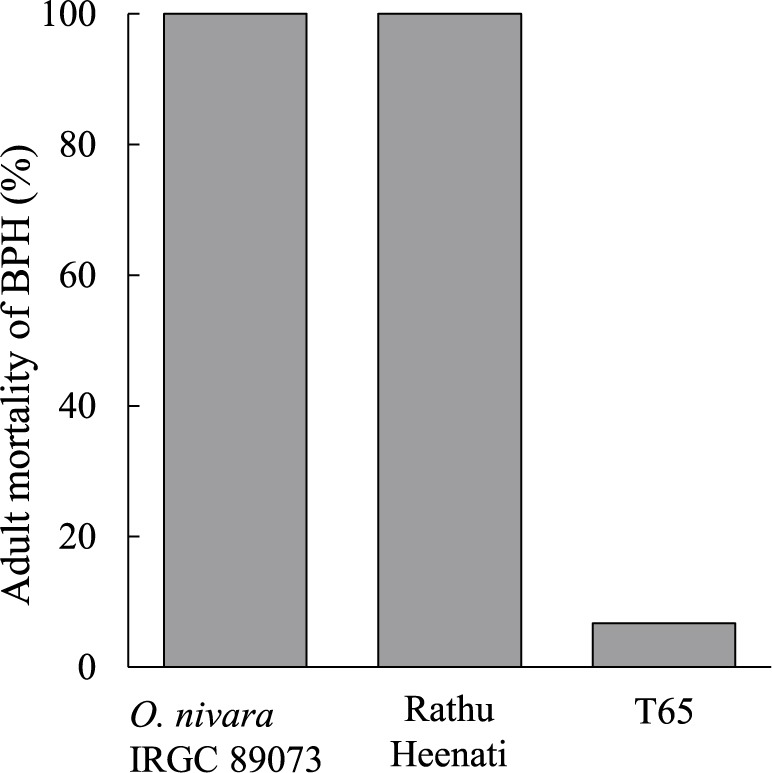
Adult mortality of BPH (%) on *O. nivara* IRGC 89073 infested by Koshi-2013 BPH population at 5 DAI.

**Fig. 3.**
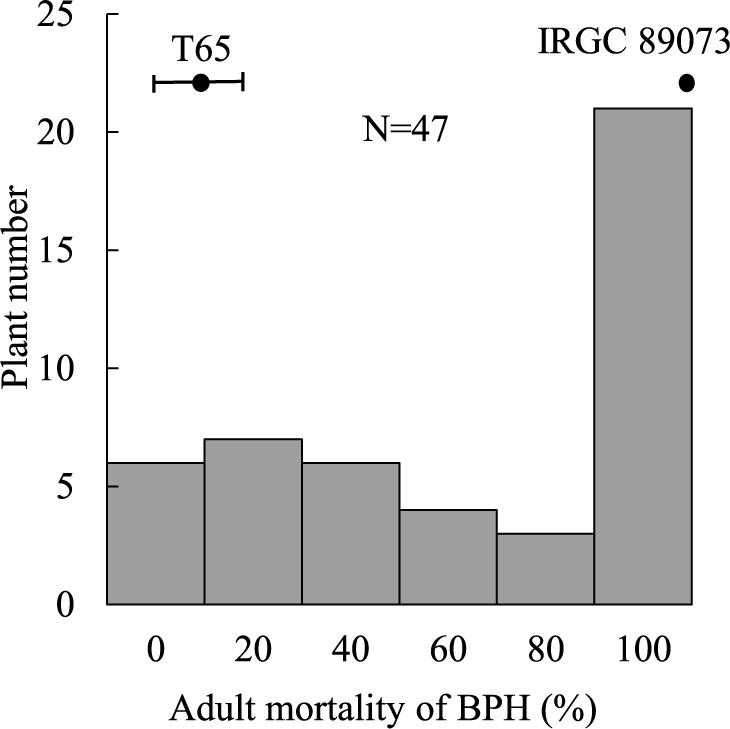
Frequency distribution of adult mortality of BPH (%) in the BC_2_F_1_ population derived from ‘Taichung 65’ × *O. nivara* IRGC 89073 infested by Hadano-1966 BPH population. Bars indicate means of parents with SD.

IRGC 89073 exhibited strong resistance to Koshi-2013 population with 100% adult mortality of BPH similar to the known resistant cultivar ‘Rathu-Heenati’ (Fig. 2). In contrast, the T65 was susceptible with 6.7% adult mortality of BPH. This significant difference between T65 and IRGC 89073 confirms the strong antibiosis in IRGC 89073. To identify the genetic basis of its BPH resistance, we evaluated the BC_2_F_1_ population by antibiosis test. The frequency distribution of adult mortality at 5 DAI was continuous, ranging from 0% to 100% (Fig. 3, Supplemental Table 2). This distribution suggests that IRGC 89073 has multiple QTLs contributing to BPH resistance. CIM identified *qBPH11* between markers RM5582 and RM5349 on chromosome (chr.) 11, with phenotypic variance explained (PVE) = 21.3%. The IRGC 89073 allele reduced adult mortality and increased BPH resistance (Table 1).

### Confirmation of QTL for BPH resistance in BC_3_F_2_-11 population

**Fig. 4.**
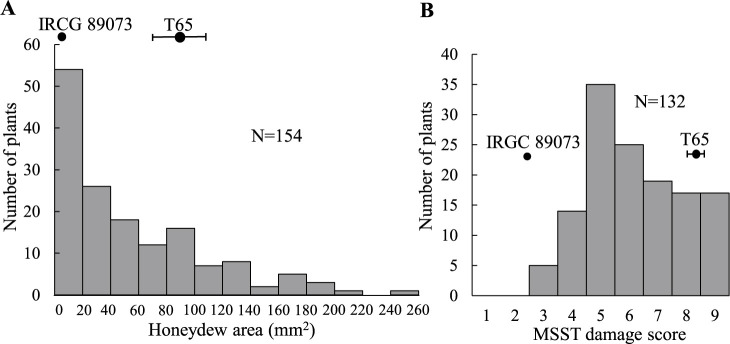
Frequency distribution of (A) honeydew area in the BC_3_F_2_-11 population and (B) damage scores in modified seedbox screening test (MSST) in the BC_3_F_3_-4 population derived from ‘Taichung 65’ × *O. nivara* IRGC 89073 infested by Hadano-1966 BPH population. Bars indicate means in parents with SD.

To confirm *qBPH11*, we conducted QTL analysis of the BC_3_F_2_-11 population. The IRGC 89073 was strong resistance with a honeydew area of 0.0 mm^2^, whereas T65 was susceptible with a honeydew area of 87.1 mm^2^. The distribution of honeydew area in the BC_3_F_2_-11 population was continuous, ranged from 0.0 to 258.9 mm^2^ (Fig. 4A, Supplemental Table 3). A single QTL, *qBPH11*, was detected between RM3083 and RM5582 on chr. 11 (PVE = 41.8%). The IRGC 89073 allele decreased honeydew area and increased BPH resistance (Table 2, Supplemental Table 4).

### Detection of additional QTL for BPH resistance in BC_3_F_3_-4 population

**Fig. 5.**
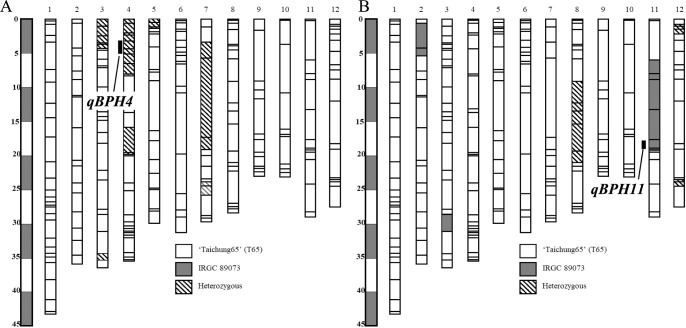
Graphical genotypes of (A) *qBPH11*-NIL at BC_3_F_3_-11 and (B) BC_3_F_1_-4 population (*qBPH4* QTL analysis). The 12 bars indicate the 12 rice chromosomes. Horizontal lines across the chromosomes indicate the positions of polymorphic SSR markers.

To detect any additional QTL in IRGC 89073, we conducted QTL analysis using BC_3_F_3_-4 populations that did not carry *qBPH11*. IRGC 89073 exhibited resistance with a low damage score, whereas T65 was susceptible with high damage score. The distribution of MSST damage scores in BC_3_F_3_-4 population was continuous, ranged from 3 to 9 (Fig. 4B). The BC_3_F_1_-4 are heterozygous on chrs. 3, 4, 5, and 7 and the genotypes of these regions on BC_3_F_2_-4 were investigated for QTL analysis (Fig. 5A). A single QTL for BPH resistance, *qBPH4*, was identified between RM8213 and RM1305 on chr. 4, with PVE = 68.7%. The IRGC 89073 allele decreased damage score and increased BPH resistance (Table 3, Supplemental Table 5).

### Development and characterization of NIL for BPH resistance

**Fig. 6.**
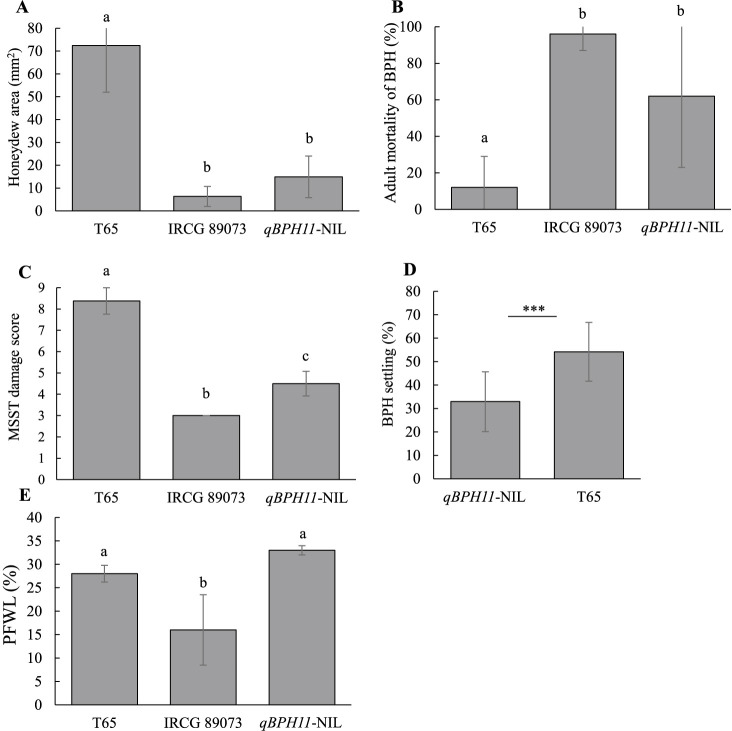
Effects of *qBPH11* in (A) honeydew test, (B) antibiosis test, (C) damage score in modified seedbox screening test (MSST), (D) antixenosis test (Hadano-1966 BPH), and (E) tolerance test (Koshi-2013 BPH). Bars indicate SD. (A, B, C, E) Bars with the same letter are not significantly different between genotypes by Tukey–Kramer multiple comparison test (*P* < 0.05). (D) Asterisks indicate significant difference between the indicated line and T65: * *P* < 0.05, *** *P* < 0.001 by *t*-test.

We selected a NIL carrying *qBPH11* with the lowest number of IRGC 89073 segments from the BC_3_F_3_-11 population (Fig. 5B, Supplemental Table 6). The NIL carrying *qBPH11* has introgressed chromosomal segments of IRGC 89073 on chrs. 2, 3, 8, 11, and 12 and size of introgressed chromosomal segment on chr. 11 was 13.4 Mb from RM441 to RM1341. To characterize the resistance mechanism, we conducted honeydew, antibiosis, MSST, tolerance, and antixenosis tests (Fig. 6). In tests using the weakly virulent Hadano-1966 population, the honeydew area was 14.9 mm^2^ on *qBPH11*-NIL and 6.3 mm^2^ on IRGC 89073, both significantly lower than the 72.4 mm^2^ on T65 (Fig. 6A). Adult mortality was significantly higher on both *qBPH11*-NIL (62%) and IRGC 89073 (96%) than on T65 (12%) (Fig. 6B). MSST damage scores were significantly lower on *qBPH11*-NIL (4.5) and IRGC 89073 (3.0) than on T65 (8.38) (Fig. 6C). And significantly fewer BPH settled on *qBPH11*-NIL (33%) than on T65 (54%) (Fig. 6D). In the tolerance test using the highly virulent Koshi-2013 population, PFWL was nearly equivalent between *qBPH11*-NIL and T65, and was significantly lower on IRGC 89073 than on T65 (Fig. 6E). The results suggested *qBPH11*-NIL conferring antibiosis and antixenosis to Hadano-1966 population. However, the NIL had no significant resistance to the Koshi-2013 population in any test, but IRGC 89073 remained significantly more resistant to both BPH populations (Fig. 6, Supplemental Fig. 1).

## Discussion

To date, at least 46 BPH resistance genes have been identified (Fujita *et al.* 2013, Yan *et al.* 2023). However, many known resistance genes have lost their effectiveness against the recent Koshi-2013 BPH population (Nguyen *et al.* 2019), and more are likely to do so. Therefore, preserving existing resistance gene resources and identifying new BPH resistance genes are necessary for the sustainable long-term control of BPH.

Many *O. nivara* accessions carry strong resistance to BPH. *BPH34*, a resistance gene identified from IRGC 104646, confers resistance to the “biotype 4” BPH population collected in Punjab, India (Kumar *et al.* 2018). Madurangi *et al.* (2010) found several *O. nivara* accessions with resistance to BPH populations collected in Sri Lanka. Here, IRGC 89073 had strong resistance to the highly virulent Koshi-2013 BPH population in antibiosis testing (Fig. 2). QTL analysis revealed two QTLs associated with BPH resistance: *qBPH4* on chr. 4 and *qBPH11* on chr. 11, and we validated *qBPH11* (Tables 2, 3).

Most identified BPH resistance genes have been detected on chrs. 3, 4, 6, and 12 (Fujita *et al.* 2013), and only three genes have been identified on chr. 11 (Supplemental Fig. 2). *BPH28*(t), from ‘DV85’, was fine-mapped between Indel55 and Indel66 (16.90 Mb–19.96 Mb) (Wu *et al.* 2014). *BPH43*, from ‘IRGC 8678’, was mapped between indel markers 16–22 and 16–30 (16.79 Mb–16.90 Mb) (Kim *et al.* 2022). *qBPH11.3*, from landrace CL48, was fine-mapped between indel markers 11M16.781 and 11M16.896 (16.75 Mb–16.90 Mb) (Li *et al.* 2024). Here, we validated *qBPH11* between RM3083 and RM5582 (16.43 Mb–17.71 Mb) (Table 2), which overlaps with *BPH28*(t), *BPH43*, and *qBPH11.3* (Supplemental Fig. 2). *qBPH11* may correspond to one of those genes. Fine mapping will be necessary to determine the exact location of *qBPH11* and to clarify its allelic relationship with known BPH resistance genes. Several other BPH resistance genes have been identified on the short arm of chr. 4 (Supplemental Fig. 2): *BPH12* from B14 (introgression line of *O. officinalis*) between RM16459 and RM1305 (5.21–5.62 Mb) (Qiu *et al.* 2012); *BPH15* from B5 (introgression line of *O. officinalis*) between RG1 and RG2 (6.90–6.95 Mb) (Yang *et al.* 2004); *BPH17* from ‘Rathu Heenati’ between RM16493 and RM16531 (6.37–7.93 Mb) (Shar *et al.* 2024); *BPH17-ptb* from PTB33 between RM1305 and RM6156 (5.62–7.86 Mb) (Nguyen *et al.* 2021); *BPH35* from RBPH660 between RM3471 and PSM20 (6.31–3.94 Mb) (Zhang *et al.* 2020); *BPH36* from GX2183 between S13 and X48 (6.46–6.49 Mb) (Li *et al.* 2019); *BPH40* from SE232, SE67, and C334 at 4.48 Mb (Shi *et al.* 2021); and *BPH41-2* from GXU202 between W4-4-3 and W1-6-3 (4.68–4.78 Mb) (Wang *et al.* 2022). In the same region, we identified *qBPH4* between RM8213 and RM1305 (4.42–5.62 Mb) (Table 3). *qBPH4* overlaps with *BPH12* (5.21–5.62 Mb) and the position of *BPH40* (cloned at 4.48 Mb), and partially overlaps with *BPH17-ptb* (5.62–7.86 Mb) and *BPH41-2* (4.68 and 4.78 Mb). Other genes, such as *BPH15*, *BPH17*, *BPH35*, and *BPH36*, are located adjacent to *qBPH4*. Therefore, *qBPH4* may correspond to those BPH resistance genes. Fine mapping is needed to determine its precise position and its relationship with these known resistance genes.

Plant resistance to insects works through three main mechanisms: antibiosis, antixenosis, and tolerance. Antibiosis affects insect survival or development, antixenosis deters insects, and tolerance allows the plant to endure damage with minimal cost (Kogan and Ortman 1978, Painter 1951). Understanding the resistance mechanisms associated with individual resistance genes is important and can be useful in combining or pyramiding genes, improving the durability and effectiveness of resistance to BPH (Du *et al.* 2020). Several genes have been reported in the same region on the long arm of chr. 11: *BPH28*(t) confers resistance mainly through tolerance (Wu *et al.* 2014), and *BPH43* and *qBPH11.3* both function through antibiosis and antixenosis (Kim *et al.* 2022, Li *et al.* 2024). Our results indicate that *qBPH11* contributes to BPH resistance through antibiosis by inhibiting BPH sucking and antixenosis (Fig. 6A–6D), but not through tolerance (Fig. 6E). This suggests that *qBPH11* corresponds to *BPH43* or *qBPH11.3*. The possible candidate gene of *BPH43* was nucleotide-binding LRR receptor (NLR) family protein and *BPH43* was associated with rapid defense activation, including the induction of defense response pathways, hydrogen peroxide catabolism and hypersensitive response (Kim *et al.* 2022). Similarly, *qBPH11.3* was mapped to a region containing a cluster of disease resistance genes and candidate genes of *qBPH11.3* were NLR family protein (Li *et al.* 2024). Based on previous study, the biological functions of NLR family proteins related to BPH resistance suggested that these genes may inhibit feeding, growth and reproduction of BPH through callose deposition and cell wall thickening (Yan *et al.* 2023). The observed phenotypic effect and its overlap with known genes suggest that *qBPH11* may function through inhibiting the sucking of phloem sap of BPH from the plant, by structural reinforcement of cell walls. While *qBPH11*-NIL showed strong resistance to BPH in different evaluation tests, agronomic traits such as yield components and heading date on *qBPH11*-NIL were not evaluated in this study. Agronomic traits for *qBPH11*-NIL will be characterized in future study. The *qBPH4* is adjacent to *BPH15* and *BPH17*, which are reported to encode lectin receptor kinases (*OsLecRKs*) involved in pattern-triggered immunity and downstream defense activation (Cheng *et al.* 2013, Liu *et al.* 2015). Additionally, *BPH40* encodes leucine-rich domains (LRDs) proteins that increase cell wall thickness, physically restricting BPH feeding (Shi *et al.* 2021). We did not directly evaluate the resistance mechanism of *qBPH4* other than MSST and it will be necessary to evaluate in detail.

IRGC 89073 proved resistant to both Hadano-1966 and Koshi-2013 BPH populations (Fig. 6, Supplemental Fig. 1). Several studies have found that highly resistant rice cultivars often possess not only major resistance genes but also minor genes, which together can enhance the durability of resistance (Hu *et al.* 2016). Here, *O. nivara* IRGC 89073 showed strong resistance to the highly virulent Koshi-2013 BPH population. *qBPH11* alone appeared to be ineffective and did not confer the same resistance level as in the donor parent (Fig. 6, Supplemental Fig. 1). These findings suggest the need to develop a pyramid line combining *qBPH4* and *qBPH11*. The combination will likely to be additive, its evidence has been demonstrated in combinations such as *BPH3* + *BPH17-ptb*, *BPH2* + *BPH32*, and *BPH20* + *BPH32*, which improved resistance against both weak and virulent BPH populations like Hadano-1966 and Koshi-2013 (Nguyen *et al.* 2019). Further research is necessary to identify the precise locations of these QTLs and to explore their potential for use in breeding programs focused on developing durable, BPH-resistant rice cultivars.

## Author Contribution Statement

NHN, NDC, and DF designed the study. NHN, NDC, and DF developed the plant materials. SSM provided BPH populations. SZ supported the research and writing. NHN, NDC, and DF performed experiments and wrote this paper.

## Supplementary Material

Supplemental Figures

Supplemental Tables

## Figures and Tables

**Table 1.** QTL for BPH resistance detected by composite interval mapping in the BC_2_F_1_ population derived from T65 × *O. nivara* IRGC 89073

QTL	Chr.	Marker interval	Physical location (Mb)	LOD score	Phenotypic variance (%)	Dominance effect*^a^*
*qBPH11*	11	RM5582–RM5349	17.7–19.0	2.5	21.3	31.1

*^a^* Positive effect indicates the effect of the allele from *O. nivara* IRGC 89073.

**Table 2.** Confirmation of QTL for BPH resistance detected by interval mapping in the BC_3_F_2_-11 population derived from T65 × *O. nivara* IRGC 89073

QTL	Chr.	Marker interval	Physical location (Mb)	LOD score	Phenotypic variance (%)	Additive effect*^a^*	Dominance effect*^a^*
*qBPH11*	11	RM3083–RM5582	16.4–17.7	17.3	41.8	–42.5	–29.5

*^a^* Negative effect indicates the effect of the allele from *O. nivara* IRGC 89073.

**Table 3.** QTL for BPH resistance detected by interval mapping in the BC_3_F_2_-4 and BC_3_F_3_-4 populations derived from T65 × *O. nivara* IRGC 89073

QTL	Chr.	Marker interval	Physical location (Mb)	LOD score	Phenotypic variance (%)	Additive effect*^a^*	Dominance effect*^a^*
*qBPH4*	4	RM8213–RM1305	4.4–5.6	33.7	68.7	–2.0	–0.6

*^a^* Negative effect indicates the effect of the allele from *O. nivara* IRGC 89073.
