# Supplementary material for: Identification and characterization of QTLs for brown planthopper resistance from wild rice, Oryza nivara (Sharma et Shastry)
Source: Breed Sci. 2025 Oct 25;75(5):369–77. doi: 10.1270/jsbbs.25038 (PMC13129573; doi:10.1270/jsbbs.25038)
Supplement: Supplementary file 1 — Supplemental Figures [file 75_369_s1.pdf]

doi: 10.1270/jsbbs.25038

## Supplemental figures

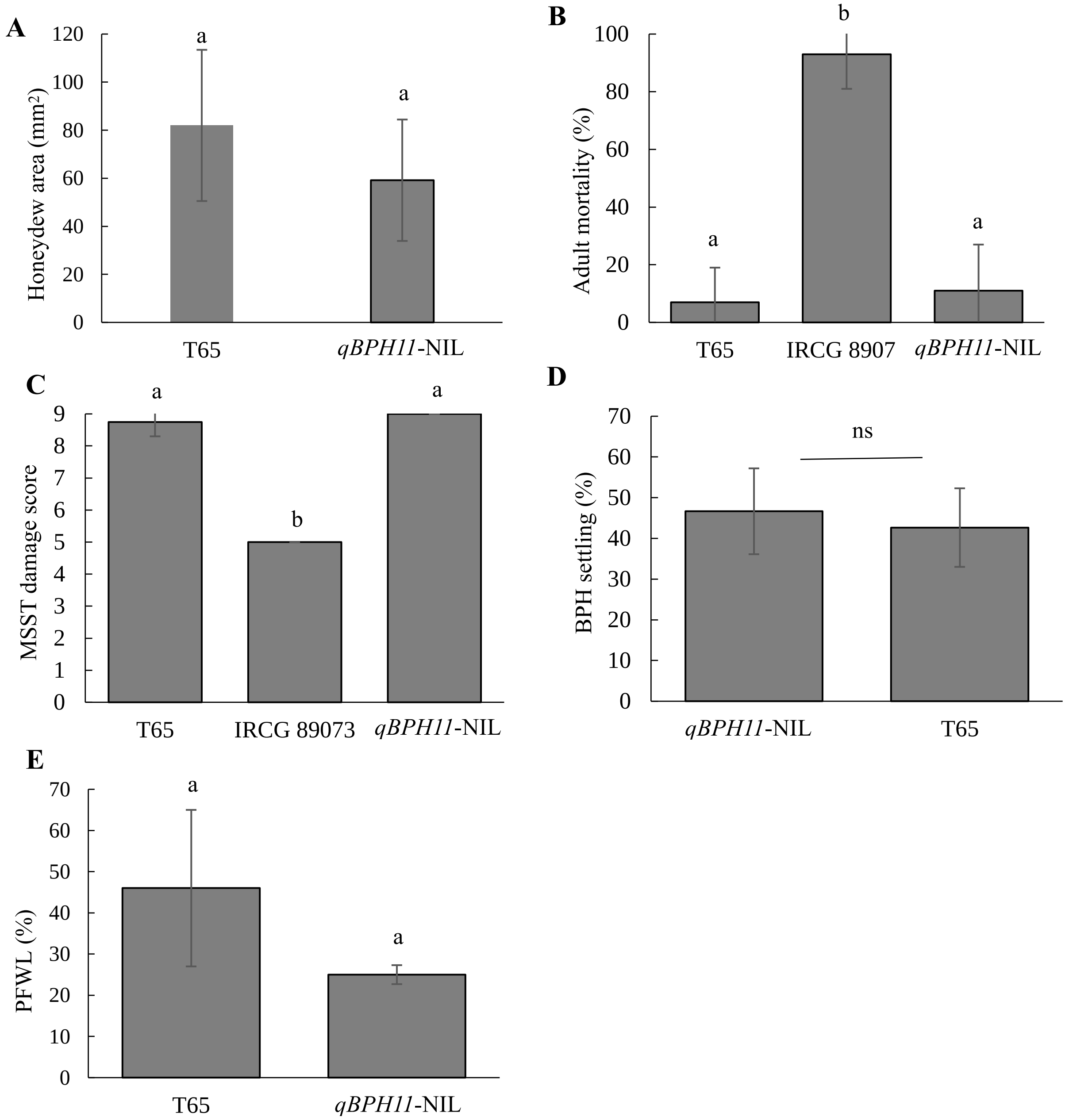


**Supplemental fig. 1**. Effects of *qBPH11* in (A) honeydew test, (B) antibiosis test, (C) damage score in modified seedbox screening test (MSST), (D) antixenosis test using Koshi-2013 BPH population, (E) tolerance test using Hadano-1966 BPH population. Bars indicate standard deviation. (A, B, C, E) Bars with the same letter are not significantly different between genotypes by Tukey–Kramer multiple comparison test ($P < 0.05$). (D) Asterisks indicate significant difference between the indicated line and T65: *$P < 0.05$, ***$P < 0.001$ by t-test.

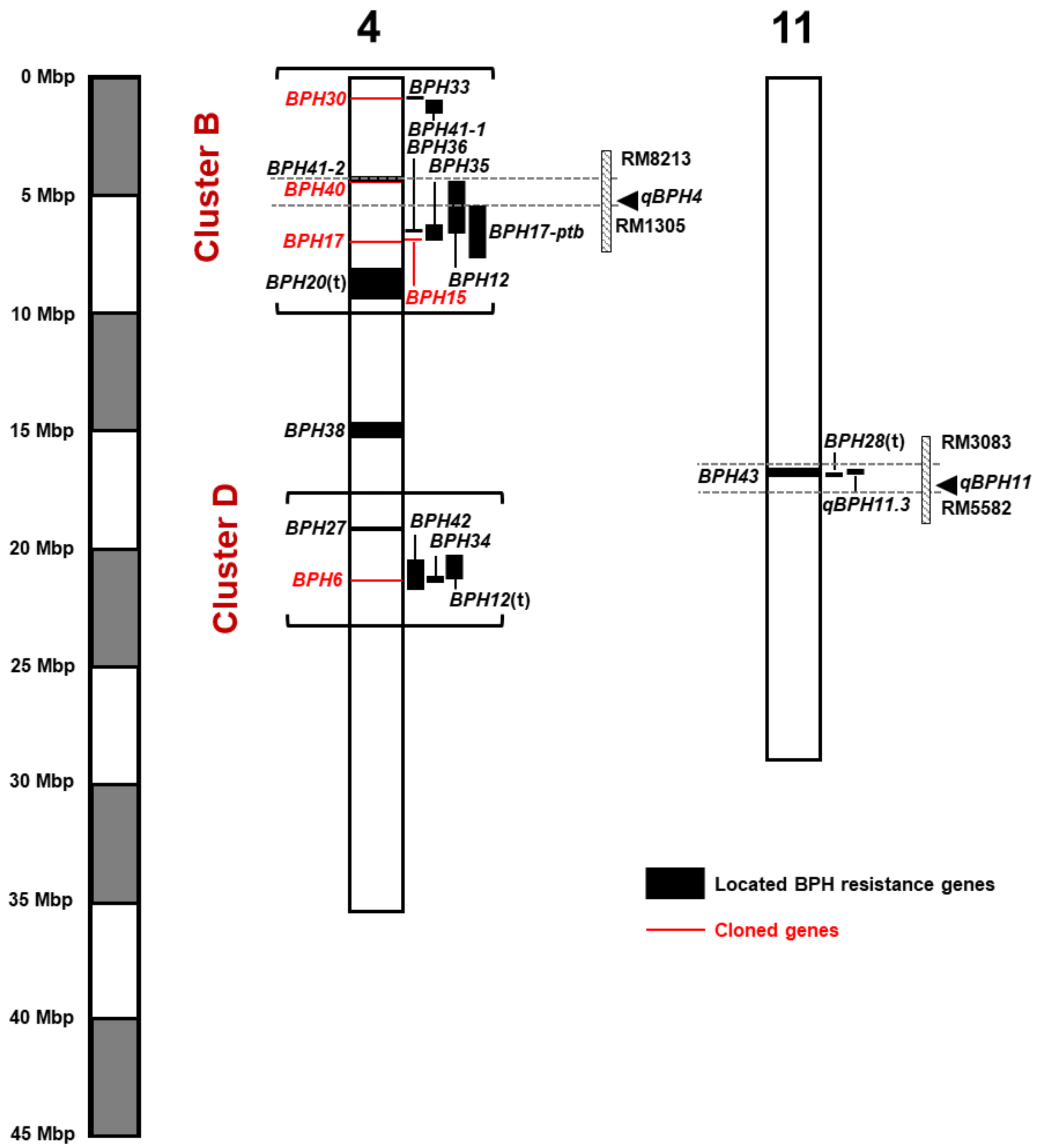


**Supplemental fig. 2.** Physical locations of brown planthopper resistance genes on the chromosome 4 and 11. QTL positions are represented as hatched bars with the LOD peaks shown by triangle and dashed lines indicate interval markers of LOD peaks.
