# Supplementary material for: Identification and characterization of QTLs for brown planthopper resistance from wild rice, Oryza nivara (Sharma et Shastry)
Source: Breed Sci. 2025 Oct 25;75(5):369–77. doi: 10.1270/jsbbs.25038 (PMC13129573; doi:10.1270/jsbbs.25038)
Supplement: Supplementary file 2 — Supplemental Tables [file 75_369_s2.pdf]

doi: 10.1270/jsbbs.25038

Supplemental Table 1. Total 384 SSR markers screened for polymorphism between IRGC 89073 and T65.

SSR marker	Chr.	Forward Primer (5’-3’)	Reverse Primer (5’-3’)	Physical location (Mb)	Polymorphic status
RM3252	1	ATGCAAGCATCTGCTTATGG	GTTGGTAACTTTGTTCCCATGC	0.30	Polymorphism
RM3148	1	GCTTTGGTATTTGCAGGTTCACG	CTATTGCTCGAACACTTTGCTTCTCC	0.75	Polymorphism
RM7278	1	GCTAGTCTGATGAATGCCGGTAGG	GCCATGTGGCAATCTGAGAGC	1.79	None
RM6324	1	CTGTACAAGAACGGCAGCAACC	GCACCACCAAACAGAGACAGAGG	2.38	Polymorphism
RM5336	1	ACCCATCACCATGGCATCAATCC	GATCCCGCGGGTTTCGTAGC	3.37	Polymorphism
RM3174	1	GCTTCAGGAGCTCTCCAAAGAGG	CTAGCTCCGTGTCAGCAACACC	5.10	None
RM8078	1	CACCGACTCCCACTATGTCTGC	AGCTCCCAACTGACAAACTCTGG	7.28	None
RM259	1	GAAGTGCTCCCTAAACTTGTTGC	TTATGGAGGATGGATTCGAAGG	7.45	Polymorphism
RM8148	1	GCCTTCACCGCTTCACCC	GTCTGCAACGCATCGAAGAAA	8.43	None
RM581	1	ATGCGTGATCAACAATCGAGAACG	CCATAGCCGATGGATTGAAAGTGG	9.11	None
RM8268	1	AAATCGACATTCTCTGTTGC	ATGGCTTACCTGCTGTCTC	9.19	Polymorphism
RM1032	1	TGTTCTTGTGGCTGAACTCTTGC	GGCACTTCACGTAGCAAACTAGC	9.32	Polymorphism
RM493	1	GTACGTAAACGCGGAAGGTGACG	CGACGTACGAGATGCCGATCC	12.26	Polymorphism
RM5365	1	CAAAGTTCGGATCATCTGTTCG	ACATATCAGGGAAAGCGTCAGG	14.50	Polymorphism
RM5385	1	ACTTTGACCTCCTTTAGCATCC	GGCCAATGTATAATCGTCTTCC	17.31	Polymorphism
RM5638	1	GGCTTCCTCATCGCCATC	CTGAGCAGCATTCCAGTCTG	20.93	Polymorphism
RM9	1	GGCCCTCATCACCTTCGTAGC	CGTCCTCCCTCTCCCTATCTCC	23.32	Polymorphism
RM8129	1	AGTACAGAACCACACAACACTG	GTCTCGCACGTTCCAATAT	25.07	Polymorphism
RM3475	1	ATGTTGTCGAGTCGTGGTAATGC	TATTCCTCGGTGTATGGGTCTCC	26.04	Polymorphism
RM11448	1	GAAGTGATCATTGGCCTGTTGC	GCATAAACAGCAGGATCCATATCAGG	26.81	Polymorphism
RM246	1	CGAGCTCCATCAGCCATTCAGC	ACTTGAGAGCGAGATTGGGAATCG	27.33	Polymorphism
RM5497	1	AGCAGACCAATGAACCATGAACC	GGTTTGGCTAAACTCATGAGAGAGG	27.54	Polymorphism
RM11498	1	TCTATTTGTCGGCGTCCCTGAGC	GAGCAGCCTGGAGTGCATGG	27.73	None
RM1297	1	AACGCAGTGCAAGTTGTATAGTGC	TCAGTACGTCCGGTCTGAAAGC	28.61	Polymorphism
RM5931	1	CTCTCCTGCCTGACAAAAGC	GTTTTAGCGGATGTGGCATC	29.28	Polymorphism
RM1231	1	GAGACCTGCCTCCCTCTACACG	GAGAGATGTACGGACGAATGAAGG	29.47	Polymorphism
RM6405	1	CTAATCTGACTGGAAACCCAGATCG	GAGCGGAGGAGCTATAGGATCG	29.53	Polymorphism
RM11668	1	AGTGTCTCTGGAGTTGGGAGTGG	CTGTTCTTCCAGATGGGCTTCC	31.42	None
RM3709	1	ATCAGCATCCCAAAGCTAGAACC	AACCGTATATTGAGGGAGCAAGC	31.94	Polymorphism
RM1216	1	ACATCGCATGCAGACTGATTAGC	CCAATGGAACAGTGACACAAGC	32.10	Polymorphism
RM1003	1	TTCCAGCTTTGCATGAACAACG	TGTACGCGACCCTGATGTCC	33.47	Polymorphism
RM7180	1	GTGTTTATAGGGGTGCCACG	TGTTGGTGGTGCAGGTAAAG	34.10	None
RM11787	1	GTCACCCTCCTTCTACATAGAGC	TACATAACGCAACAGACAGACG	34.34	Polymorphism
RM8084	1	GCGCCCAATGCATGTAAATTCC	TGCCGATACCTGTGATCAAGTCC	34.90	Polymorphism
RM5389	1	TGTGCTATTGCGCGAGATTATCC	CATCACCGCTCCAACTCATGC	35.73	Polymorphism

RM11908	1	AGCGGCAACAGTGTCGTCTCG	GCCAATGAGGCAGCAACAGC	37.18	None
RM11935	1	AGCTGACGCCTGGACTATGAAGC	CACAGCCTAAGTCTCTGTCAACATCC	37.78	None
RM8231	1	GCGTAAGATCTCCCTACCAC	CAACACATGATAGCACATGG	38.19	Polymorphism
RM7246	1	CATCACTGATCACTCAGTCACCTAGC	ATCTCCATTTCTCTCCGATGAGC	39.16	None
RM5536	1	CACGTACCAGCCTTGATGAATCC	TGGGCTATACTAATCCCGTCATCC	41.16	Polymorphism
RM5310	1	GGGACCAAGACCTTTCCAATGC	GCGGAAGCAGGAGAATCGTAGC	41.19	Polymorphism
RM6321	1	CCCAGGAATATCTCTATACACACAGC	CTCTACCTCGCTGTTGTCTCTGC	42.92	Polymorphism
RM3362	1	ATTGCGTGGGATATGGACTATGC	CCTCTTCAATTCCCTTCCTCTGC	43.04	Polymorphism
RM7451	2	TTGCAGCTTGTGTCGTTTACTGG	GCATTGGCGTTTGTTAATCACC	0.65	Polymorphism
RM2770	2	AATCAACCTCTGATCCCAAGTTCC	TTAGCTGCTGATCTGCCCTATCC	0.81	None
RM4355	2	GGGATGAGAGTAGAAGGCACAAGG	GCTTAATGCCTTTGATCGTTGC	4.26	Polymorphism
RM1347	2	AGCTGCAGCCACATGATCAGC	CGGCACCAGCTAGTAGAATTAGTTGC	5.31	Polymorphism
RM5862	2	CCTCCTGAAGGGTAAAGGATTGG	TCCACACATGATCGCTACATCG	6.01	None
RM7636	2	ACAGTCACTAACACAGGCAGAGC	CCCAAGCTAGTACCAACTCAAGG	7.66	Polymorphism
RM145	2	CCGGTAGGCGCCCTGCAGTTTC	CAAGGACCCCATCCTCGGCGTC	7.71	None
RM6853	2	TGCATTTCTTGCCACAGGTAGG	TCAACACGCACATCCTGTACCC	8.96	Polymorphism
RM6639	2	ACCGGAAGGGATACTTATCAGC	CTTCCTGTGAAATAGTAGAGGTAGGC	9.42	None
RM1313	2	TTTATTCCGTCCAAGCTGTTCG	GGGTGATTTGGCTAAACTATGTGC	11.26	Polymorphism
RM324	2	CTGATTCCACACACTTGTGC	GATTCCACGTCAGGATCTTC	11.39	Polymorphism
RM1190	2	TTGGATTGCAGGAGGAGAAGG	CAATCCAAGCCGTAAATACGAACG	12.09	None
RM5812	2	CGCTGACATCTTGCCCTC	GTAGGACCCACGTGTCATCC	16.00	Polymorphism
RM262	2	CATTCCGTCTCGGCTCAACT	CAGAGCAAGGTGGCTTGC	20.80	Polymorphism
RM5427	2	TGCTGTTGACACTTGACAGGTAGC	CACAATTATTGCGGCTCATCG	21.54	Polymorphism
RM5651	2	AAGAGAACATTTGGAATCCT	TAACCTGTGCCTTGTTTG	23.59	None
RM3515	2	CATGCTAGTAAGCAAAGGGCAACG	TTGCACGTCCAACTGTCCAAGC	24.04	Polymorphism
RM3515	2	CATGCTAGTAAGCAAAGGGCAACG	TTGCACGTCCAACTGTCCAAGC	24.04	Polymorphism
RM1920	2	GCCTGGTAAGTGGTAATGTAATGG	GTGAATTCCTCCTTGGTCTTGG	25.49	Polymorphism
RM3730	2	CCACTGATACAGAAGCTGATGAGG	AGCACAATGACTCCCTTCTTTGC	26.26	None
RM5631	2	CGTCCAAGAAATATTGCAGT	GTGAGACAGAATCCTTACGC	28.29	Polymorphism
RM6	2	GTCCCCTCCACCCAATTC	TCGTCTACTGTTGGCTGCAC	29.59	None
RM5472	2	CACTCAAGACCAGACCTGTACG	CGGCACGTCATTGTAGTGAC	30.66	Polymorphism
RM240	2	CCTTAATGGGTAGTGTGCAC	TGTAACCATTCCTTCCATCC	31.50	None
RM017	2	TGCCCTGTTATTTTCTTCTCTC	GGTGATCCTTTCCCATTTCA	32.49	Polymorphism
RM016	2	CGCTAGGGCAGCATCTAAA	AACACAGCAGGTACGCGC	32.49	Polymorphism
RM5916	2	GCTATAAGAATCGTATTAAG	TACTGCTATTAAAGTCAGAA	34.08	None
RM3692	2	TCTCTTGCTGGTCTCCGTCTTCG	AAGAAGAATCGGCGGATTAACAGG	34.66	Polymorphism
RM3248	2	GAACCTTGTGATATGGTTACGC	GGAGCTAAGGTATAGTAGCAACAGC	35.05	None
RM3894	3	CGCACTTGCTTAGAAGTCAATCATCC	ATGCTCTCTCCTTCAGGCCATCC	1.10	Polymorphism
RM3372	3	CAAAGAATCCAAGGAGGCCAAAGC	ATGCCGATGAGCACCCAGAGG	1.44	None
RM231	3	CCAGATTATTTCCTGAGGTC	CACTTGCATAGTTCTGCATTG	2.45	Polymorphism

RM3807	3	ACACGGGCACACTCTTTTTC	GTTGAGCTTTTGGCCTATGG	3.51	Polymorphism
RM3126	3	CCTCCTTCGTCTTCTCCTTTGC	CTCCGGTACGTCGAAACTCG	3.66	Polymorphism
RM489	3	GAACAGGGACACAATGATGAGG	GACGATCGGACACCTAATTACAGC	4.32	Polymorphism
RM489	3	GAACAGGGACACAATGATGAGG	GACGATCGGACACCTAATTACAGC	4.32	Polymorphism
RM1278	3	GAATTCAAAGGGCTCACTTCTGC	CCATATAAAGGTGGCACGACAGG	4.54	Polymorphism
RM4992	3	CCATGACTGTCTTGTTCAACTTCAGC	TCGGTGGTGCTACCTCATAATCC	4.71	None
RM5755	3	CCATGGTCGCCATTGACACG	CCTGCTATAACACTCGCACAGATGC	5.93	Polymorphism
RM3872	3	GGCTCACCAAATTAAGAGCTTGC	TGATGATGCTTGCCTTAGTGTCC	6.82	None
RM3766	3	CGCGTTCGATCGATCTCTCTCC	GGCCAGAGTACGTGCCAGATGC	6.90	Polymorphism
RM1022	3	GTCTTTGATAGCGGCTTTGTCC	GGATGAGGGAGTAATGTCTCTTGG	7.20	Polymorphism
RM3461	3	CCACCCTTTGTTCTGAGTCTATTGC	GTCGAATGGCATGAACGTAAAGC	8.32	None
RM251	3	GAATGGCAATGGCGCTAG	ATGCGGTTCAAGATTCGATC	9.95	Polymorphism
RM3280	3	CGAATATTCAGGTTGGAGCAAACG	CGATTGGTCGCTCTAGCTTCTGG	10.87	None
RM282	3	CTGTGTCGAAAGGCTGCAC	CAGTCCTGTGTTGCAGCAAG	12.41	Polymorphism
RM5903	3	TGTCTAGGTTCGTAGCATGTGTTCC	CCACTGAAAGCACCGTTAAATCC	13.25	None
RM3291	3	TCCTATACTTGTCTGTCCATCGATCC	GTTCCTGCACAACAACAACAACC	13.57	Polymorphism
RM6594	3	ATCGTGCAGCAGGTCATCTGG	CGGGCAATTTCTCATCACTCAGC	13.93	None
RM6676	3	AGAGGAGAGATACAGATTGAGACG	GACCCTTGATGTGAGTAGTTGG	14.33	Polymorphism
RM6676	3	AGAGGAGAGATACAGATTGAGACG	GACCCTTGATGTGAGTAGTTGG	14.33	Polymorphism
RM6959	3	GATTCCTATGGAGGATTGTTGC	AACTCCACCGGTGTTAAGAAGG	14.34	Polymorphism
RM3204	3	CTTACACACATGGCCCACATGC	TCCTCTCTTCTCACTCTCCCAACC	14.83	Polymorphism
RM6881	3	CGACTGATTCGATTCCACAATGATCC	GCAGCTGGAAGCAGAGGAAGACG	16.69	Polymorphism
RM3180	3	CGCGGAGTTGAATTCTGAACC	GGGTCGGATAGCCACACACG	18.07	None
RM5684	3	ATTCTGACGCACCAGTACCCTACG	CACATCGAGGATCTGATTGAACTGG	18.21	Polymorphism
RM411	3	GTAGGAAATTCTTCGCCAGATGC	CCGAGACTTGGAACAATCTTAGGC	21.23	None
RM5864	3	AGGGAGTAACTAACAAGGTCACAAGG	GCATAAATTCAGCTCCGTACAAGG	22.19	Polymorphism
RM8208	3	GCCCAAACTACACTCTCTTG	GTAAATGCCTGAGTGCCTAC	22.20	Polymorphism
RM8208	3	GCCCAAACTACACTCTCTTG	GTAAATGCCTGAGTGCCTAC	22.20	Polymorphism
RM16	3	GTGCGCCAGGAGTAGTTGTCTCC	GACGTGTACACATAGCCAAATCATCC	22.93	None
RM6266	3	CACCTTCTTGAGAAGCTCCTTCG	GACATCGAGAGCGAGGACAGC	23.62	Polymorphism
RM2334	3	CATGCATCTGATCTGATTAT	TGTGAAGAGTACAAGTAGGG	26.55	None
RM3436	3	AGAGTTGTTAGCATGGCAGCATCC	CAATTGGCCATTGCAAACATGG	27.22	None
RM168	3	TGTCGTCGAGGATTTGGAGATCG	GAATCAATCCACGGCACAGTCC	27.90	Polymorphism
RM7117	3	GTTGGCTGGTTGCTACCACTACACG	AGCTCCGACTCTTCGCCTTGC	28.58	None
RM6329	3	CCCTGGATGAAAAGCACAAG	GAAGTTGTAGATGCCCCATC	28.61	Polymorphism
RM8203	3	CATTGATAATGTCCAGTGACG	CTCCTGTTGTCATTCTTTGG	31.19	Polymorphism
RM3684	3	CGACGCAACCTATACTTGACAAAGG	GGTGTCCCAGCAAGTATTTCACC	34.41	Polymorphism
RM570	3	GTTCTTCAACTCCCAGTGCG	TGACGATGTGGAAGAGCAAG	35.38	Polymorphism
RM1221	3	GTGGGCAGATTATGGTACGATGC	AAGCATGTCCCTGCAACTTTAGG	35.46	None
RM7324	3	GAGAGAGAGAGAGGAGAGGCG	GATGCACATCTCGACAGCTC	36.13	None

RM551	4	CTTACTCCATTGGGCTGGAACC	TGTAGGGTGGTAAGAGATCCACTCC	0.17	Polymorphism
RM335	4	GTACACACCCACATCGAGAAGC	TCCATGGATATACGAGGAGATGC	0.68	Polymorphism
RM518	4	CTCTTCACTCACTCACCATGG	ATCCATCTGGAGCAAGCAAC	2.02	Polymorphism
RM5414	4	ACCATGGTTCAAGAGTGAAA	ACAGCTCAACCTGTTGAGTG	2.02	Polymorphism
RM5414	4	ACCATGGTTCAAGAGTGAAA	ACAGCTCAACCTGTTGAGTG	2.02	Polymorphism
C61009	4	GGCCAGCAAGGTGTAGTAAG	ACAAACCCCAGCACCCTAAG	2.43	Polymorphism
RM8213	4	TGTTGGGTGGGTAAAGTAGATGC	CCCAGTGATACAAAGATGAGTTGG	4.42	Polymorphism
MS5	4	TTGTGGGTCCTCATCTCCTC	TGACAACTTGTGCAAGATCAAA	7.25	None
MS10	4	CAATACGAGAAGCCCCTCAC	CTGAAGGAACACGCGGTAGT	8.07	Polymorphism
B40	4	CAATACCGGATATCTTGACTCC	CGACCACGCTGCCTATATTC	8.21	Polymorphism
RM307	4	GTACTACCGACCTACCGTTCAC	CTGCTATGCATGAACTGCTC	13.15	None
RM401	4	GCATGAGCTGCTCTCATTATTGTCC	GAAACGAACCAAACGTTCATCG	13.21	Polymorphism
RM5900	4	TTCTACGTTTGACCGTCA	TCTAGGAGCGTTTGTAGGAG	13.83	Polymorphism
RM5687	4	AGAGAAGAAGGGAAGGAGGAAGG	AGTGACTTGTGGGTGACTTGTGG	15.93	Polymorphism
RM5687	4	AGAGAAGAAGGGAAGGAGGAAGG	AGTGACTTGTGGGTGACTTGTGG	15.93	Polymorphism
RM6314	4	CATGTCTGATATTGCGGTTCAGG	TCAAGCCCTGCCCAACTACG	18.63	None
RM471	4	AGAAATGGATCGGACTGAACATGC	AGACACTCGGACGCACAAGC	19.01	None
RM1205	4	CAATCACAGAGCAACACGTACCC	GCAGAGGCAGCTGAGAAGTATAGC	19.64	Polymorphism
RM5586	4	AGATGGCTGGCCAACAGACTGG	ACAATGCCCATCCACTGCTTCC	19.91	Polymorphism
RM1359	4	CGACTTGCCAAAGGTCAACG	GATTCTACGGGCCACAAGTCC	20.04	None
RM1359	4	CGACTTGCCAAAGGTCAACG	GATTCTACGGGCCACAAGTCC	20.04	None
RM5749	4	GCTCGTTTCTCTCGATCACTCG	GCAAGGTTGGATCAGTCATTTCG	20.13	Polymorphism
RM1155	4	GACAGGGAGTGTGGCAACTATGC	GATCACAGACAATCATGGGTTGG	20.53	None
RM1155	4	GACAGGGAGTGTGGCAACTATGC	GATCACAGACAATCATGGGTTGG	20.53	None
RM6997	4	CGGCAGTAAATTTGCATTGACC	AGTGGCCTTGTCAGTCTACATGC	21.28	None
RM3839	4	ATGCATGTGATGCCAAGAGTGG	GAAAGCACACTGCACACATACCC	24.07	Polymorphism
RM3785	4	GCAAGCAGCAAGAGCGAAGAGG	CTCAAGGCCGCCTCTCAAATCC	24.22	None
RM1136	4	AGAACCACTCATTGGAGCTACGC	AGCTGTAAATGTTCAGGGCAACC	25.20	Polymorphism
RM252	4	TTCGCTGACGTGATAGGTTG	ATGACTTGATCCCGAGAACG	25.36	None
RM7187	4	CACACCAACTTCTCGCGGTAAACG	CACAGCGAACGTGGTGTCTTCG	27.62	None
RM3916	4	CGATGTAGGTAGAGGGACACC	GTCGTACGTCCAGATCTAGTCG	28.73	None
RM3781	4	ATTTAGGGTTGGTGCTGCAG	AGTTGGGCAACAGATCCATC	28.76	Polymorphism
RM6365	4	GAGGAGAGGAGAAGGGAGAAAGG	GTGTTGCACCTCCTACCTCTGG	29.81	Polymorphism
RM6480	4	AATCCCTCGAGGCGCAGAAGC	CCACAAGGCTCACCTCGATGTCC	29.85	Polymorphism
RM7208	4	GCCAATGTGTGAGAAGAGGATAGG	GAGGCAAGCTTTCTACCATTATGC	30.18	None
RM5503	4	GGAAGGGAAGAAGATAGGAGATGG	CTAGCATAGGCCTCCAACAAACC	30.40	Polymorphism
RM3276	4	ACAGGTCGATCTCGATGAACTCC	CTCTTCTCCGTCTCGACTCTTCC	30.72	Polymorphism
RM3276	4	ACAGGTCGATCTCGATGAACTCC	CTCTTCTCCGTCTCGACTCTTCC	30.72	Polymorphism
RM5030	4	AGATTTTAGTGGTCCAAACA	ACTCAATTTCAACAATGGTG	30.75	None
RM5511	4	GAGTTCGTCCCTGACAAACAGAAACG	GTGAGCGAGCGAGTGAGTGAGC	31.15	Polymorphism

RM3534	4	TTGAGCTTCGTCTACAAGCG	CAGCTCCCACCATCTCTCTC	31.20	Polymorphism
RM1250	4	GAAACCACGACTAGGCATCG	CTTCCACAAGGTCTCGCTTC	31.26	Polymorphism
RM3687	4	GGGCCCTACTAGTACAGCTAAAGG	AATTGGGAAGGAAGGAACAGG	31.52	None
RM3843	4	CCAGATCATCCAGGCATAACATCACC	CGGCGCTGGTAAACTCCATTCC	31.72	Polymorphism
RM3836	4	CGGAATCACCAATTTCTCTCTCAGC	CGCAAGAAACGGAAACGAAACC	31.85	Polymorphism
RM6909	4	AAGTACTCTCCCGTTTCAAA	CCTCCCATAAAAATCTTGTC	32.09	Polymorphism
RM348	4	CATGAAGCTGTGTTGCTGTTGC	CGCTACTAATAGCAGAGAGACCATCG	32.87	None
RM3335	4	CCTTACGCACCATCATCTTGTACC	CATGGATGGCTCACTAGTTATTGC	33.06	None
RM1113	4	GTTCTTGGGTTGGTGAGCTTCC	TAGGGCGCATGTGTATTTCTTCC	34.30	Polymorphism
RM6006	4	GTCAGCTCGGCGATGAACAGC	TCCATTCCATCGTTACCGTCTCC	34.93	Polymorphism
RM5879	4	ACCATTAAGCCTTGTACTCAGC	TAACCAGAGATCGATTGGTAGC	35.15	None
RM1272	4	CAGCAACAATGCTCCCTCTCC	CAGTTGCAGCATGATGTCTATGG	35.33	Polymorphism
RM6320	5	CGCTACGGAAGGGTAATAATGC	AGCGTGGGAAGAAGGATACACC	0.44	None
RM3796	5	ATTAGCCTTTAATTCCACTG	ATACAAACAAACAGCTTGTG	0.46	None
RM7029	5	CTTGATCAGTTAAAGAGCCAGTCTGC	TTCAAGGCAGACAACAACATGG	0.54	Polymorphism
RM5796	5	AGGCGATGGAACATGAAGTGTGC	AGAGCCGGAATGTTCTCCTTTGC	1.14	None
RM1024	5	AACTGCCATCTCTGAAACTCTGC	CATCTCACTTCAGAAGGATCATAGCC	1.17	Polymorphism
RM5374	5	TTAGGGTTAGGTCTGTTGTGTACTGG	CGCTGGATCACAACACTGTAGC	1.20	None
RM6317	5	GGAGACAGTGGAGAGGCTACTGG	CATCATCAACTACCAACCCATCC	1.46	Polymorphism
RM5579	5	CAACAGAACCTGCCAACTCTTGC	TGCCTCATGGTAATAAGCCAAGC	1.86	Polymorphism
RM413	5	CCAATCTTGTCTTCCGGATCTTGC	AGATAGCCATGGGCGATTCTTGG	2.15	Polymorphism
RM413	5	CCAATCTTGTCTTCCGGATCTTGC	AGATAGCCATGGGCGATTCTTGG	2.15	Polymorphism
RM405	5	TATGCTTTCTGTCAGCTTCC	CTGCTGTGAAAGAGTTGACG	3.01	None
RM3777	5	TGATCTCACCTCTTCCCTACAAGC	GCTCCCAAATCTTGGTCTTGC	4.11	Polymorphism
RM4691	5	CCATCAAGAGATAGTGCTCCAACC	ACACAGCCTATGTTTAGGGTTTCC	6.94	None
RM169	5	CACCTCCTCCAAGATCCTTATGC	CTCTCTGTCTCGCTGTCTGTTGC	7.40	None
RM7293	5	CACATGGTCACATGGTGTGTAGC	GAGTAGTTCCTGCCGAGCAACC	7.43	Polymorphism
RM289	5	TTCCATGGCACACAAGCC	CTGTGCACGAACTTCCAAAG	7.81	Polymorphism
RM5844	5	AACGTGGCATCCATGTTAGTACC	AGCTAGGAGCCATTGTCGAAGG	9.05	Polymorphism
RM3381	5	ACGAACGCGAGCTGACAGAGG	AATAGCTGCCAGCAACTGCAACG	9.49	None
RM249	5	CAACTCCACTCCAGACTCAACTCC	GGTATGATGCCATGAAGGTCAGC	10.68	Polymorphism
RM6645	5	CTCCGGGATGCCATAGTTTCG	AAGCTTCCTCTCGATCGTCTTCG	14.94	None
RM3838	5	GTTGGTAGTGTCTTTGTGCAAGC	GCAACACCTCTTTCAATCTTCC	16.42	Polymorphism
RM3437	5	CCAAGACGCTCAAGATCTGC	AGCCTTAGCAACGAGGTTATTGG	16.42	Polymorphism
RM1237	5	CAGCACACATACTCTGGCTCTCC	CCGCGAGCTTTAGAAGAGAAGG	17.87	Polymorphism
RM430	5	GTCCCTGATCAGAAACGAGATGG	TAGGGTTGGAAGAATGCAAGACC	18.61	None
RM163	5	CGCCTTTATGAGGAGGAGATGG	AAACTCTTCGACACGCCTTGC	19.11	Polymorphism
RM3351	5	GTCGAAACGTAGCCAGGCAATGG	CCATGGAAGGAATGGAGGTGAGG	20.62	Polymorphism
RM4244	5	GATTAATTTTCACATGAATA	GAATTTGTAAACTTAATGAA	21.93	None
RM6841	5	CTTCCCGAAATCAGATTCTTGC	CGACGAGTCCTACACACTCTCC	22.66	Polymorphism

RM7081	5	CTTCCCGCACTACACTGCACTCC	CTGCAACTTGCTCATGGAGTTGG	24.44	None
RM6545	5	GTGATGGTTGCTTTCTTTCTCTCG	TCACGTTGCTCAGCTCACTATCC	24.79	Polymorphism
RM7446	5	CGTTGAGCCAAGAAGAAGAAAGG	TTTGAAGGCAGTTTCACTGACG	24.82	None
RM3348	5	CTTCTCGGTTCATCCAAAGAGC	GTGGAAGCTATGGGTAGCTCACG	24.95	Polymorphism
RM3170	5	GCAGTGTCATTCTCATGAAACCTACC	CAGACTCCAAAGCACCCATAACC	27.80	None
RM6360	5	ACGTGGAATCCAAATTGACAGC	TTCGCTGCACTGTTTACTCTTGG	27.92	Polymorphism
RM6313	5	ACACTATCCAGATCCACTTTGACC	CATCCTTGAGTTTGGCTTTGG	28.12	Polymorphism
RM31	5	CGCTCCTCCACTCTTCTCCTACC	CGTGCAGAAAGTCCATTACTCTCC	28.45	Polymorphism
RM6775	6	AATTGATGCAGGTTCAGCAAGC	GGAAATGTGGTTGAGAGTTGAGAGC	0.21	Polymorphism
S00310	6	CAACAAGATGGACGGCAAGG	TTGGAAGAAAAGGCAGGCAC	0.21	Polymorphism
MSSR1	6	CTAGCTGCTCTGCTCTGCTG	CGGCAATCTCTCCGAATC	0.22	Polymorphism
RM508	6	AGAAGCCGGTTCATAGTTCATGC	ACCCGTGAACCACAAAGAACG	0.44	None
RM469	6	TTACGTGATCACACAGGCTCTCC	AAGCTGAACAAGCCCTGAAAGG	0.56	Polymorphism
RM589	6	GTGGCTTAACCACATGAGAAACTACC	TCACATCATTAGGTGGCAATCG	1.38	None
RM586	6	TGCCATCTCATAAACCCACTAACC	CTGAGATACGCCAACGAGATACC	1.48	Polymorphism
RM1369	6	CATCGATTAGCTTACATGGCAACG	ACTAGTGCGACCGTCTTCAATGG	1.56	Polymorphism
RM190	6	CTTTGTCTATCTCAAGACAC	TTGCAGATGTTCTTCCTGATG	1.76	Polymorphism
RM204	6	GTGACTGACTTGGTCATAGGG	GCTAGCCATGCTCTCGTACC	3.17	Polymorphism
RM225	6	TGCCCATATGGTCTGGATG	GAAAGTGGATCAGGAAGGC	3.42	None
RM1163	6	TGGACGCGGATAGGAGGAGACG	TCCTCCGCAAGGTCGGTTTCC	4.20	Polymorphism
RM217	6	ATCGCAGCAATGCCTCGT	GGGTGTGAACAAAGACAC	4.24	None
RM314	6	CTAGCAGGAACTCCTTTCAGG	AACATTCCACACACACACGC	4.85	Polymorphism
RM253	6	CCATCTCTGCCTCTGACTCACC	TCCTTCAATGGTCGTATCTTCTCC	5.44	Polymorphism
RM2615	6	ATCTCGTTCATACTGCTTGACC	GACTGGTTTCCTTCATGTTACC	5.97	Polymorphism
RM276	6	GTCCTCCATCGAGCAGTATCAGC	CTAGCAAGACATGGACCTCAACG	6.24	Polymorphism
RM8274	6	TCTCCTCTGCATCATTTCC	CTACTCCCATCCTGTTCCTC	6.58	Polymorphism
RM549	6	ATCCCTGAACCCAAATTCTGTCG	CTCTTTGATCTTCCGGTGATTTCG	6.99	None
RM7488	6	GTGTTCGGAGATGAGAGGATACG	GCGCTTTCTATTGGAGATGAACC	7.01	None
RM5585	6	TCAGAGGTGGCAGCTTATTTTATACC	ATGTAAATGGTCACACACACACACAC	7.62	None
RM8240	6	TGATTGGTGATAATTGGAGAG	ACGAGGTTCTCGAGATGG	8.88	None
RM6836	6	TGTTGCATATGGTGCTATTTGA	GATACGGCTTCTAGGCCAAA	9.32	None
RM527	6	CGGTTTGTACGTAAGTAGCATCAGG	TCCAATGCCAACAGCTATACTCG	9.87	Polymorphism
RM7311	6	CTAGTTTATGCCCTCGTTTCTTGC	ATGGAAGTGGTCGTTGAACTCG	10.89	Polymorphism
RM3330	6	CGTTCGAGCAGAACCATCTACC	CCTCTTCCGCTCCACTCTCC	10.91	Polymorphism
RM3183	6	GTGGTGCTAGTATGGACGAGAGG	CGGTTGGTAGACTGTAAACAAAGTGC	12.29	None
RM003	6	GAGCCGAACTGACCTCCACTGC	CACTGCCTCCGGGTCATTGC	19.15	None
RM7193	6	ATGTGGGAATTTCTAGCCCC	CCCTAGTTTTCCAAATGGCC	19.91	Polymorphism
RM3827	6	TAGGTAGGACCGTGCTTCATTGC	CCCTGGCCTTTCTTCAATCTGC	21.95	None
RM6395	6	GGCTTCGGCTTCTGAACTAGC	CGACTAAGCAGCAGTAACAATCTCG	25.61	Polymorphism
RM400	6	TTACACCAGGCTACCCAAACTCG	TTGCTGAGTTCCCTCGTCTATCC	28.05	Polymorphism

RM3138	6	GTGGTGAATGTTGAGCTGCATGG	GACTGAGCCAAGTTGCTGTCTGG	28.09	Polymorphism
RM1370	6	GGAGGGAGGAATGGGTACACG	TTGAGAGTGAAACGAGAACCAACC	28.15	Polymorphism
RM340	6	GGGTAAATGGACAATCCTATGG	ACCCTATTCTGGAGTTCATCTGG	28.22	None
RM3343	6	ATCAAGGCGGTACAGGCACACG	CGCAGATGGCTGAAACCCTAACC	29.10	Polymorphism
RM7454	7	CCTCCTTTCTCACTCACCTTCTATCC	TTGGCCATTTGATCGTGATAGG	1.10	None
RM6697	7	TATTCCCGGGAGATCCAACAGC	AAGATCCAGTCGATTTGGTTCAGG	1.19	Polymorphism
RM481	7	TAGCTAGCCGATTGAATGGC	CTCCACCTCCTATGTTGTTG	2.88	None
RM1353	7	ATTAAGCTACTGTCTGCCTCCTTCG	GCTTCGTTTCAGGTGGTCAGG	3.35	Polymorphism
RM1243	7	CACCTCAACCGATAGAGTGAACC	TCTATTTCGGGAGGGATTATGACC	3.59	None
RM6728	7	GGGTATGTGTCGCTATTTTA	GAAATCTGGAATTTTCCCTA	5.76	Polymorphism
RM1377	7	ATTAGATACATCAGCGGGGG	GCTGCTGTACGATGTGATCC	12.73	None
RM214	7	CTGATGATAGAAACCTCTTCTC	AAGAACAGCTGACTTCACAA	12.73	None
RM021	7	ACAGTATTCCGTAGGCACGG	GCTCCATGAGGGTGGTAGAG	13.41	Polymorphism
RM5481	7	CAAGTTTGGCAGCTAAATGTCTCC	GGTTGCACAGAGTAGTGATGTTTCG	16.15	None
RM6767	7	GGTACAAGCAACTTGCACTGTGG	CATCGCCTGGGAGATCATACG	17.42	Polymorphism
RM5793	7	TGGACACAACACATTCCATCTCC	TCAGCTTTCTTCTTTCTCCCAAGC	17.44	Polymorphism
RM11	7	ATCGGTGCTTGGCTGGATAGC	CCACCTTCTTCTCCTCCTCTTCC	19.20	Polymorphism
RM3404	7	CTCCTCAGTCCTGAGTCTCCTGTCC	CCCAGAGAGATTACACAGAGCAAGC	20.05	Polymorphism
RM3799	7	CCAGGGAACAAGTATGCGATCC	GGAGATGTCTTATCAAGGTCACTTGC	21.58	Polymorphism
RM5508	7	TCTTCTCCATCCAGAGACAATCC	ATGCCGTCTCGCACACTAGC	23.51	Polymorphism
RM5847	7	CTTTAGGTAGCGTCATCTTCC	TGGAAATACAGAAGGAGTCG	23.60	Polymorphism
RM1132	7	TCAAGGTCGACATGTTAGGTATGC	AACCCTATCACCTGAGAAACATCC	23.93	None
RM7040	7	AGTAGTGTTGTGTGATCAGTAGGG	GAGTATCTACGTACGGATGTCTGC	23.96	Polymorphism
RM6098	7	GCCGCCATGAGCAACAGAGC	TGGCTGCGAGGAAGAAGAACTAGC	24.41	None
RM505	7	AGAGTTATGAGCCGGGTGTG	GATTTGGCGATCTTAGCAGC	24.53	Polymorphism
RM234	7	TTCAGCCAAGAACAGAACAGTGG	CTTCTCTTCATCCTCCTCCTTGG	25.42	None
RM5426	7	CCATACGACTCCACAACACACTGC	AATCGCAAGCGGATCGAAAGC	25.88	Polymorphism
RM5455	7	GATCAACGAACCCACCACACC	CGCGTCTTGTATATGCACTTGATCC	26.41	None
RM1306	7	AGTCTCAAAGGCATACAGTACACACC	GCCAATTACCTTCCCGTACATAGC	28.89	Polymorphism
RM248	7	AGAGAGCAAGTTTGAAGCGAAGC	ACCAAGAGGGTAGCCTAGCATGG	29.29	Polymorphism
RM5911	8	CCCTCTTTTTAAGTCTGGGG	GGTGCCTCCTTTCAAAGTTG	0.07	Polymorphism
RM6369	8	CAAGCTAGGGCTGCATAAGC	GCTTCACCTACCTACCTCACC	0.12	None
RM152	8	GAAACCACCACACCTCACCG	CCGTAGACCTTCTTGAAGTAG	0.68	Polymorphism
RM3309	8	GCCTACTCAGCTTCCTCTCCTTCG	CGCCATTTACGGCAGCAACC	1.19	None
RM6356	8	GAGACTTGGCGACTCTGATCTGC	TGATCTCCTCCTCCTTCGTCTACC	1.56	Polymorphism
RM6863	8	CTACTGCCTCATTGCTCACATGC	TCAGGCAAGAACAGTTGATGAGC	2.01	None
RM1148	8	CATGCTCGAATCAGTGAGTAGTGG	GCTTAGCTATGCAGGAGAGAGTGG	3.73	Polymorphism
RM3572	8	CCATTTGGTAGGTCCATCTTACCC	CTCCCAAGTGAAGTGCTGTCTGG	3.92	Polymorphism
RM025	8	GGAAAGAATGATCTTTTCATGG	CTACCATCAAAACCAATGTTC	4.38	None
RM5556	8	GTAAGCCATTTGCACGGACAAGG	GAGCTCAGGATCATCCCTACATGC	4.58	Polymorphism

RM7057	8	TCAGCACTCACAGAAGTCTAATCACG	GTCGTTGATCGATCGAGTAGTGG	5.85	Polymorphism
RM6429	8	GATGTTGGTGTGCAGGGTGTAGG	GTCACCACCCTCTACGTACGTTCC	8.38	None
RM3481	8	CCTCACGTCGTGCTCTCCAACC	CCTCGTCGCGTTCGTCAACC	9.13	Polymorphism
RM331	8	ATGTTGCACTCCTTCAATGTCC	CATGAGACAATGCCAGAAAGC	12.29	Polymorphism
RM3383	8	CCTACCTCTAGCGGCTGACTGC	CCATCAATGCCGGTAGTTCG	13.48	Polymorphism
RM404	8	GGAGCAGCTAAGGCAGATAAGAGG	GCCTTCATGCTTCAGAAGACAGC	15.43	Polymorphism
RM3689	8	CGTCAGCCGAAACTACTATCTAAACC	GTTTCACTGCACTCTGGTTTGC	19.33	Polymorphism
RM5887	8	ATTTCAGGGTTACAGCCCTACC	AAATGCCACATCTATCCCTACC	20.97	Polymorphism
RM284	8	ACTGCATGATCCTCCTCAGATCC	CCCTCTGATCTCTGATACTCCATCC	21.14	None
RM5985	8	CAGAGTAAGACCCGGAGCCTGAGC	AGGAAGGACCTGGGCGTCAACC	21.58	Polymorphism
RM556	8	AAACTAGTACTATGCCGCTACTGC	CATGTACTCCAAACCTCACTGC	22.34	Polymorphism
RM6976	8	CTGCAACCTGCACGAGTACACC	GTCCCATTGGATAGAATCCCAGAGC	23.55	None
RM308	8	GGCTGCACACGCACACTATA	TTACGCATATGGTGAGTAGGC	24.79	None
RM1345	8	CGCACAAACCAAACACACAACC	CTCGTGTCGTCTTCACCAAAGC	26.14	None
RM1615	8	AAACGTAGACGAAGATCACCTGTGC	TTCGAGAGTGATCAGTACCCTTCTCC	26.26	None
RM502	8	CATCTCTGTTCCACTTGCTTTGC	CTACCAACAACCCAACAAGAAGG	26.49	None
RM3761	8	TGTGGAGTGTGGAGGCGAGAGC	AGGCACCCACCCACTGACTTCC	27.10	Polymorphism
RM6966	8	ACCAACAAATGGGTCGTATTGG	CTACGGATTTACCCAGAAACATGG	27.32	None
RM6845	8	CGGCAAACTCTACGAGGTAATGC	CACACGTTCTTCTCCTTCATCTCG	27.56	Polymorphism
RM3120	8	GGAAGCTCTTTGCCATCATACTAGC	GAATATAACACCCACTCCCTCTGTCC	27.81	Polymorphism
RM3496	8	GGTATACGGCCCTTCAAGTACACG	TGCAGAGAAAGAAGGGAAGATCG	27.83	Polymorphism
RM3155	8	TGCGTGTAACTGTTTCGCTTGC	CCCTTGAGCTGATGCACATACG	27.90	Polymorphism
RM5688	9	GGTGATGATGAGTGTTTGATGC	TGACAGTAGTAGTTCAGCAGTGTCC	1.66	Polymorphism
RM444	9	TGCATCTTTCACCGTAGTCCTAGC	CTTGCTGGAGCTCGTAGATGC	5.87	None
RM1328	9	GAATGGGATTAGACGATTTG	CCATGAGTGACATCAAAAGG	9.15	Polymorphism
RM7390	9	TGAGAGCTCGTAGGAAGTGTCC	CAGAGTCAGCAATCGCTAAGG	10.42	Polymorphism
RM3769	9	CTGAAATCTGTGAAAGCCTGAACG	GCTGGTGACAAACTGCATCTTCC	11.69	Polymorphism
RM7039	9	CTTCCAGTGAGGTGACTCTACGC	ATGCACATTTGCCATTCTACCG	14.63	None
RM7424	9	CAGATCAAGCTAGCCACACAGC	GAAGGCAGAGCAGGAGAGAAGC	16.53	None
RM7048	9	CGTGAAAGTGACGAGTTTCAGTCC	CGAAGTGAACATGGCACAAACC	16.88	Polymorphism
RM6543	9	AGCGGGCTCCTGAACAGTCTACC	CCATGCAAGAACGCGATCACC	17.70	Polymorphism
RM242	9	AAACACATGCTGCTGACACTTGC	TTACTAGATTTACCACGGCCAACG	18.64	Polymorphism
RM3164	9	TCCTCCTGCTAGCTGCCTAG	TCGCCTTCCTTTTCACTCAC	19.58	Polymorphism
RM3808	9	CAGTGGCGTGGAGAGAAATTTGG	CTCACCTGCGACAGCAAGATCG	20.25	Polymorphism
RM1553	9	TTATTGTCCATGCGGTACAACG	CAACCACCTCATCACCAACTCC	20.70	None
RM215	9	GAGCAGCAAGAGCAGCAGAGG	CATGCTCGACTTCAGAAGCTTGG	20.89	None
RM6971	9	GGACCACGCGAGGTATGAGC	CATAATAACGCGGATCAGCTAAGAGC	21.59	Polymorphism
RM7306	9	AATCAATCGCGGTTCCAGAGACG	CCACCAGAACGCGCACAAGC	21.91	Polymorphism
RM2482	9	CGGCTCAATGACAACTAAACAGTAGC	GCAGGGAATCATGAAACAGAATGG	22.28	Polymorphism
RM205	9	CTGGTTCTGTATGGGAGCAG	CTGGCCCTTCACGTTTCAGTG	22.42	Polymorphism

RM7492	10	CAAGGATTTGAGGGAACATGG	TTTAGAGATGGTTGCCAAGAGC	0.03	Polymorphism
RM5271	10	CGAATCTTGGAACACATCAACG	GGGAGGAGTGCTGTGAGAGG	1.92	None
RM7545	10	GTTTCCATATCCGTGCTATTCG	CACGATTCCTACAATACGAGAGC	3.79	Polymorphism
RM216	10	GATGGTAAAGGAAGAACGTGTGC	CACTCATAGACGCATCACATAGCC	4.99	None
RM5348	10	TCACCGAATCCGATAGGAGTACC	CCTAAAGTGTATGGGCTGGAATGG	8.08	None
RM6833	10	TAGGACTCGATCAAGGGAATTGG	TTCCTTCCTTTCTCTCCCTTTACC	10.89	Polymorphism
RM467	10	TGTTGTCACATGAGATGGCTATGC	GCTGACCTTGTGAGACGTTTAGACC	13.04	None
RM5806	10	GAATGCTAATTGCGGTTGAAGC	GGATCTTTCCTCCCAATCTTTGC	14.04	None
RM1375	10	GCTTGTTGGCAGTTTGTATTGTGG	GGTACACTAAGTGGGCAAATCAGG	16.20	Polymorphism
RM5620	10	GGGCCGATTTGTCTTTCTTTCC	CTGTCGACTTGAAGCATCACACC	16.96	Polymorphism
RM1125	10	AATGAGAGTGGGAAGAACACATCC	GGGACACACTCAGAAGTGAGAGC	17.33	Polymorphism
RM1873	10	CCAGACAAGCAACACCTACTTCC	CAAACAGGCCCTAACTGACAGG	17.38	None
RM5274	10	GGAGAGAGAAGAGAGGAAATGACG	CTCATGTTGGAGAAGAGTTTCTACCC	17.40	None
RM5629	10	CAACAACATCTGTGAGGGTTTCG	CACCACCATCTCCTCTTTCACC	18.23	None
RM3123	10	ACGCTCTTAATTGATCCGTTCG	CAAAGTCCAGTTCCGTTGATCC	21.32	Polymorphism
RM496	10	GACATGCGAACAACGACATCC	CTATAGTTGTTGCACATGCGATCC	21.98	Polymorphism
RM4771	10	AACTGAGAAACATGGGACAGAAGC	CTCAACCACACCCTCATTCACC	22.57	Polymorphism
RM590	10	CATCTCCGCTCTCCATGC	GGAGTTGGGGTCTTGTTCG	22.59	Polymorphism
RM1761	11	ATCTTACACTGTGCAGGTTGTGC	GGAACTTCCTTGGTGAATGAGC	0.31	Polymorphism
RM1240	11	CACTCACATGCTCAAATGGATCG	CACCTGATCAAGCCATATCACTCC	1.46	None
RM6544	11	ACACCGTGAATGCTCTGCTTCG	ATGCACCCTTCGTCACAGAAGATCC	3.84	None
RM167	11	CTCCGAGTCCGACCACAAGG	TCCAGCCCTTCCTATCATATTGC	4.06	None
RM5704	11	TTGATCACCTACACGTACACATGC	AAGGTCCTTTGAGCATTCAGTCG	5.40	None
RM441	11	AAGGGAGTAGCCTCTCCATCTCC	GTGCTGACTCCTCTCCCTGTCC	6.01	Polymorphism
RM3701	11	GAAAGAGGAGGAAGAGCTAGAGG	CCATATGTACGGAGTGTGTTTACC	8.02	Polymorphism
RM536	11	TACCAGGATCATGTTTCTCTCC	ACTGTGAGATTGACTGACAGTGG	8.89	Polymorphism
RM5731	11	CTCCACCCTAAGCTTCTCCTGTGG	GCGCATCCATCCCTCTTCACC	9.86	None
RM6091	11	GCGGACACACCAGAGAATAAGC	GTGCTGTCCTGTCCTTGAATCC	13.29	Polymorphism
RM3428	11	GCCATTGACACCAAATGATCACC	GGCATATAAGGTCCATGGTGAATTGG	13.37	None
RM3083	11	CCAAGCTTAAAGTTATTCCA	ACCCAATTTGAATCATGTAG	16.43	Polymorphism
RM5582	11	AGGCGTGTGTGTGTCTTTTG	GAAACTCTGTACCTCCCTTGTG	17.71	Polymorphism
RG2	11	TTCACCAACCCTTCTCCTC	ACTTGGCAACCTCTTTCATC	18.32	None
RM229	11	CACTCACACGAACGACTGAC	CGCAGGTTCTTGTGAAATGT	18.87	Polymorphism
RM5349	11	CATCCAAATGTTGCGGATTACC	TTCAATAGCCCAGAGAACCAAGC	18.99	Polymorphism
RM5961	11	GATCAGCAGTGGACGATTCACC	TCTCCTGTATGCTCCTCCTCACC	19.22	Polymorphism
RM1341	11	AACCTGGAGGTGCTGGTCTC	TTTCTCCCCCCCAACCAC	19.44	Polymorphism
RM1219	11	CGACGAGGAATGGAGGAGTTTGG	TCCTATGTTTCAAACCTGCCCTCACG	20.66	Polymorphism
RM4112	11	CACTGGCAAAGTCAGTAGTCCTTCC	GGAGGCTAGCTCCGTAAATCTGG	24.26	Polymorphism
RM7240	11	AAGCGACGACGAAGCTACCTACC	GGTGATCAGACAACGAGCTTCC	26.17	None
RM224	11	ATCGATCGATCTTCACGAGG	TGCTATAAAAGGCATTCGGG	26.80	Polymorphism

RM6094	11	CGATTGCGATGGCGATTAGG	GAATCGGTGGAAGAGGTGACG	28.30	Polymorphism
RM5926	11	TAGGTCCATCCAAATCTCGATCC	TGGCAGAGGAGATTAGAGTAATACGG	28.33	None
RM1880	12	TCACCAGGGTCGTTAAAGTACTGC	GCAATACCACATCTGATCCACACC	0.75	Polymorphism
RM3323	12	CATCTTGGCGTTGATACGAAGG	ACGAGTCGAGCACTACATCTCTCG	0.98	Polymorphism
RM3483	12	CCTAGCTTTCAGGAGCAAG	CCCACAATGAGAAACAGTTG	1.61	Polymorphism
RM7315	12	GCAGGAACAACAACAACAAAGG	GGCGTGTGGGTTAGTATGTACTCC	2.18	Polymorphism
RM247	12	AAGGCGAACTGTCCTAGTGAAGC	CAGGATGTTCTTGCCAAGTTGC	3.19	Polymorphism
RM7619	12	TCTTGGTATGTATTGGCAGCGAAAGC	AGGATGTGAATGAAGGCGAATGG	4.83	Polymorphism
RM7003	12	CTCTAGCTCTCTCATGGATGG	AATCATAGGGCAGACATACAGC	6.78	Polymorphism
RM3103	12	CTGGAGTGGAGAAGAGAGAACAGG	TCTCCGCTCGGTTTCATCTAGG	7.46	Polymorphism
RM1036	12	CTTCCCTGTCCATAGATCTGCTTGG	GAATGACCCAACCTGTCATTCTTAGC	8.80	None
RM101	12	AAGTAGTGGTCGAAGTGTGTATCG	GGTGAATGGTCAAGTGACTTAGG	8.83	Polymorphism
RM1337	12	AGTGGCCCGAACCTGTATAACC	GAGCAGGTGCAATGCTGAGG	12.04	Polymorphism
RM1261	12	ATGGTAGAGACACAAGTCCATGC	GACAAATTGGTGTAGGTGAAGG	17.58	None
RM1261	12	ATGGTAGAGACACAAGTCCATGC	GACAAATTGGTGTAGGTGAAGG	17.58	None
RM277	12	CGGTCAAATCATCACCTGAC	CAAGGCTTGCAAGGGAAG	18.29	Polymorphism
RM1246	12	GGCTCACCTCGTTCTCGATCC	CATAAATAAATAGGGCGCCACACC	19.16	Polymorphism
RM313	12	TGCTACAAGTGTTCTTCAGGAC	GCTCACCTTTTGTGTTCCAC	20.87	None
RM1986	12	TCTGTGGAGAAGAAATGGATCTCG	CATCTCTCCTCTAGGCGGATTGG	21.28	None
RM309	12	CACGCACCTTTCTGGCTTTCAGC	AGCAACCTCCGACGGGAGAAGG	21.52	None
RM309	12	CACGCACCTTTCTGGCTTTCAGC	AGCAACCTCCGACGGGAGAAGG	21.52	None
RM4589	12	GTTTAAACATGGGAGGTGTCAACC	CGGGCATCACTGAAATACCG	22.06	None
RM3726	12	TACACCCACCCACATACGTCAGC	GTCGTACTCCCGGATCTTCTTCC	23.31	Polymorphism
RM1103	12	GTCGGTGTGTACTCCGTGTTTGG	CATATGCAGTGGTCAGTGGAGTGG	23.61	Polymorphism
S12091B	12	GGCTTTCTTCCTCACACTGC	CGAGGACGAGATGAGACGA	23.69	Polymorphism
S20103	12	GTTCGAGGGTAACCCGAA	TCATCGCCTCGATCACAC	23.74	Polymorphism
RM5479	12	CTCACCATAGCAATCTCCTGTGC	ACTTCGTTCACTTGCATCATGG	24.45	Polymorphism
RM3739	12	CTAAGATCCAACGGGTTCTGTGC	TTGTGTGCACTTCGTCTTCAACC	25.04	None
RM6189	12	TCTCGCCTATTTACCATACTGC	AACCCTCAACTGGAATTACACC	26.06	None

Primer sequence information was obtained from: Temnykh *et al*. (2001) and McCouch *et al*. (2002), The physical position of marker was the physical location of forward primer for each marker obtained from The Rice Annotation Project Database (Sakai *et al*. 2013) based on the 'Nipponbare' genome sequence. Chr.: Chromosome.

Supplemental Table 2. Adult mortality (%) of brown planthopper (BPH) at each SSR marker position based on genotypes in BC_2F_1 population.

Marker	Chr.	Physical location (Mb)	Adult mortality of BPH (%)	
			T65 homozygous	Heterozygous
RM5638	1	20.93	58%	67%
RM6405	1	29.53	62%	63%
RM1003	1	33.47	66%	58%
RM5472	2	30.66	56%	70%
RM3692	2	34.66	58%	66%
RM3684	3	34.41	60%	64%
MS10	4	8.07	59%	68%
RM5985	8	21.58	67%	58%
RM3155	8	27.90	66%	62%
RM242	9	18.64	53%	72%
RM1553	9	20.70	56%	71%
RM6971	9	21.59	55%	65%
RM7492	10	0.03	56%	72%
RM496	10	21.98	55%	72%
RM441	11	6.01	68%	59%
RM536	11	8.89	49%	72%
RM3428	11	13.37	49%	71%
RM5582	11	17.71	44%	77%
RM5349	11	18.99	46%	76%
RM1341	11	19.44	46%	78%
RM4112	11	24.26	63%	-
RM3483	12	1.61	56%	75%

Supplemental Table 3. The honeydew area data for all individuals tested in BC_3F_2 population for *qBPH11* confirmation, including the donor and recurrent parent.

Plant no.	Entry	Honeydew area (mm^2)
1	RDG14-4	25.08
2	RDG14-5	104.92
3	RDG14-8	58.28
4	RDG14-9	28.68
5	RDG14-10	83.23
6	RDG14-11	99.86
7	RDG14-12	16.12
8	RDG14-14	99.93
9	RDG14-15	23.52
10	RDG14-16	125.09
11	RDG14-17	42.33
12	RDG14-18	51.83
13	RDG14-19	16.96
14	RDG14-20	25.47
15	RDG14-21	38.86
16	RDG14-22	32.74
17	RDG14-27	32.77
18	RDG14-32	16.96
19	RDG14-33	33.57
20	RDG14-34	72.62
21	RDG14-35	81.68
22	RDG14-37	87.73
23	RDG14-38	26.16
24	RDG14-39	67.72
25	RDG14-40	19.94
26	RDG14-41	41.55
27	RDG14-42	144.62
28	RDG14-43	199.26
29	RDG14-44	0.00
30	RDG14-45	168.53
31	RDG14-46	86.59
32	RDG14-47	30.92
33	RDG14-48	89.32

34	RDG14-49	115.58
35	RDG14-50	55.32
36	RDG14-51	107.91
37	RDG14-52	101.93
38	RDG14-53	30.99
39	RDG14-54	1.85
40	RDG14-55	13.64
41	RDG14-56	79.84
42	RDG14-57	37.26
43	RDG14-59	258.87
44	RDG14-60	42.34
45	RDG14-61	21.33
46	RDG14-62	117.63
47	RDG14-66	58.36
48	RDG14-67	7.15
49	RDG14-68	72.61
50	RDG14-72	0.00
51	RDG14-73	138.10
52	RDG14-74	162.58
53	RDG14-75	67.00
54	RDG14-77	0.00
55	RDG14-78	0.00
56	RDG14-79	128.45
57	RDG14-80	21.30
58	RDG14-81	98.79
59	RDG14-82	0.52
60	RDG14-83	15.04
61	RDG14-84	0.00
62	RDG14-85	217.55
63	RDG14-86	0.00
64	RDG14-87	1.00
65	RDG14-88	2.47
66	RDG14-89	0.00
67	RDG14-90	26.34
68	RDG14-91	0.00
69	RDG14-92	5.93
70	RDG14-93	0.50
72	RDG14-95	0.00
73	RDG14-96	0.31

74	RDG14-97	42.55
75	RDG14-98	0.13
76	RDG14-99	1.69
77	RDG14-100	30.90
78	RDG14-101	48.21
79	RDG14-102	0.00
80	RDG14-103	9.20
81	RDG14-104	25.07
82	RDG14-105	4.73
83	RDG14-107	81.97
84	RDG14-108	13.63
85	RDG14-109	65.14
86	RDG14-110	10.83
87	RDG14-113	32.75
88	RDG14-115	166.78
89	RDG14-116	39.17
90	RDG14-118	98.48
91	RDG14-120	2.77
92	RDG14-121	79.22
93	RDG14-122	110.81
94	RDG14-123	23.71
95	RDG14-124	72.44
96	RDG14-125	138.94
97	RDG14-126	9.64
98	RDG14-127	159.03
99	RDG14-129	24.79
100	RDG14-130	42.33
101	RDG14-131	190.67
102	RDG14-132	3.93
103	RDG14-133	182.47
104	RDG14-134	40.93
105	RDG14-135	0.45
106	RDG14-137	130.45
107	RDG14-138	51.59
108	RDG14-139	2.24
109	RDG14-141	54.22
110	RDG14-142	120.89
111	RDG14-143	97.28
112	RDG14-145	88.21

113	RDG14-146	31.20
114	RDG14-149	78.20
115	RDG14-150	6.66
116	RDG14-151	2.64
117	RDG14-153	0.00
118	RDG14-155	23.44
119	RDG14-156	118.13
120	RDG14-157	35.19
121	RDG14-158	74.37
122	RDG14-160	0.60
123	RDG14-163	45.81
124	RDG14-164	40.54
125	RDG14-165	54.87
126	RDG14-166	92.99
127	RDG14-169	38.93
128	RDG14-171	1.00
129	RDG14-172	28.51
130	RDG14-173	0.00
131	RDG14-174	7.45
132	RDG14-175	121.26
133	RDG14-176	0.15
134	RDG14-177	56.18
135	RDG14-178	57.54
136	RDG14-179	0.00
137	RDG14-180	11.49
138	RDG14-182	0.00
139	RDG14-183	0.00
140	RDG14-184	1.40
141	RDG14-185	1.24
142	RDG14-186	90.12
143	RDG14-187	4.30
144	RDG14-189	6.67
145	RDG14-190	0.00
146	RDG14-191	169.71
147	RDG14-192	161.69
148	RDG14-193	11.07
149	RDG14-194	93.67
150	RDG14-195	1.99
151	RDG14-196	64.45

152	RDG14-197	14.79
153	RDG14-198	124.32
154	RDG14-200	86.23
Recurrent parent	T65-1	112.40
Recurrent parent	T65-2	157.65
Recurrent parent	T65-3	105.47
Recurrent parent	T65-4	38.11
Recurrent parent	T65-5	44.05
Recurrent parent	T65-6	64.85
Donor parent	IRCG 89073-1	0.00
Donor parent	IRCG 89073-2	0.00
Donor parent	IRCG 89073-3	0.00
Donor parent	IRCG 89073-4	0.00
Donor parent	IRCG 89073-5	0.00

Supplemental Table 4. Phenotypic and genotypic data of the resistant recombinant around the *qBPH11* region on BC_3F_2-11 population.

Entry		*qBPH11* region				Honeydew area (mm^2)
	Marker	RM3083	RM5582	RM229	RM5349	
	Chr.	11	11	11	11	
	Physical location (Mb)	16.43	17.71	18.87	18.99	
RDG14-137	(S-Control)	1	1	1	1	130.5
RDG14-72	(R-Control)	2	2	2	2	0.0
RDG14-98		1	3	3	3	0.1
RDG14-179		2	3	3	3	0.0
RDG14-173		3	2	3	3	0.0
RDG14-77		3	3	2	2	0.0
RDG14-82		3	3	2	2	0.5
RDG14-171		3	3	2	2	1.0
RDG14-54		3	2	2	2	1.9

Genotype: 1 indicates T65 homozygous allele, 2 indicates IRGC 89073 homozygous allele and 3 indicates heterozygous allele.

Supplemental Table 5. Phenotypic and genotypic data of the resistant recombinant in the *qBPH4* region on BC_3F_2-4 population.

Entry		*qBPH4* region				MSST damage score
	Marker	C61009	RM8213	RM1305	RHD3	
	Chr.	4	4	4	4	
	Physical location (Mb)	2.43	4.42	5.62	6.60	
DRI 3 2-1	(S-Control)	1	1	1	1	9
DRI 3 4-2		3	3	3	1	5
DRI 3 8-7		2	3	3	1	5
DRI 3 9-8		3	3	1	1	5
DRI 3 1-2	(R-Control)	2	2	2	2	3
DRI 3 2-10		2	2	3	3	4
DRI 3 3-9		2	2	3	3	3
DRI 3 4-4		2	3	3	3	5
DRI 3 4-7		3	3	2	2	4
DRI 3 2-3		3	2	2	2	3
DRI 3 3-3		3	2	2	2	4
DRI 3 6-4		3	2	2	2	5

Genotype: 1 indicates T65 homozygous allele, 2 indicates IRGC 89073 homozygous allele and 3 indicates heterozygous allele.

Supplemental Table 6. Polymorphic SSR markers between T65 and IRGC 89073 on substitution chromosomal segments of *qBPH11*-NIL.

Marker	Chr.	Physical location (Mb)	Genotype
RM7451	2	0.65	IRGC 89073 homozygous
RM4355	2	4.26	IRGC 89073 homozygous
RM1347	2	5.31	IRGC 89073 homozygous
RM7636	2	7.66	T65 homozygous
RM168	3	27.90	T65 homozygous
RM6329	3	28.61	IRGC 89073 homozygous
RM8203	3	31.19	IRGC 89073 homozygous
RM3684	3	34.41	T65 homozygous
RM7057	8	5.85	T65 homozygous
RM3481	8	9.13	Heterozygous
RM331	8	12.29	Heterozygous
RM3383	8	13.48	Heterozygous
RM404	8	15.43	Heterozygous
RM3689	8	19.33	Heterozygous
RM5887	8	20.97	Heterozygous
RM5985	8	21.58	T65 homozygous
RM1761	11	0.31	T65 homozygous
RM441	11	6.01	IRGC 89073 homozygous
RM3701	11	8.02	IRGC 89073 homozygous
RM536	11	8.89	IRGC 89073 homozygous
RM6091	11	13.29	IRGC 89073 homozygous
RM5582	11	17.71	IRGC 89073 homozygous
RM229	11	18.87	IRGC 89073 homozygous
RM5349	11	18.99	IRGC 89073 homozygous
RM5961	11	19.22	IRGC 89073 homozygous
RM1341	11	19.44	IRGC 89073 homozygous
RM1219	11	20.66	T65 homozygous
RM1880	12	0.75	Heterozygous
RM3323	12	0.98	Heterozygous
RM3483	12	1.61	Heterozygous

RM7315	12	2.18	Heterozygous
RM247	12	3.19	T65 homozygous
RM277	12	18.32	T65 homozygous
RM1246	12	19.16	Heterozygous
RM1103	12	23.61	Heterozygous
S12091B	12	23.69	Heterozygous
RM5479	12	24.45	Heterozygous

Primer sequence information was obtained from: Temnykh *et al*. (2001) and McCouch *et al*. (2002), The physical position of marker was the physical location of forward primer for each marker obtained from The Rice Annotation Project Database (Sakai *et al*. 2013) based on the 'Nipponbare' genome sequence. Chr.: Chromosome.
